# Expression patterns and prognostic value of key regulators associated with m7G RNA modification based on all gene expression in colon adenocarcinoma

**DOI:** 10.1186/s12876-023-02657-y

**Published:** 2023-01-21

**Authors:** Yuanchang Zhu, Zeyi Zhao, Mya Thandar, Junhao Cheng, Pan Chi, Shenghui Huang

**Affiliations:** 1 Department of Colorectal Surgery, Fujian Medical University Union Hospital, No. 29, Xinquan Road, Gulou District, Fuzhou City, Fujian Province China; 2 Training Center of Minimally Invasive Surgery, Fujian Medical University Union Hospital, Fuzhou, Fujian China

**Keywords:** m7G, Colon adenocarcinoma, Bioinformatics, Prognostic signature, Nomogram

## Abstract

**Background:**

N7-methylguanosine (m7G) is present in a wide variety of organisms and has important roles. m7G has been reported to be involved in multiple biological processes, and recent studies have reported that changes in RNA modifications result in tumor cellular transformation and cancer, such as colon adenocarcinoma, lung cancer, and intrahepatic cholangiocarcinoma. However, little is known about the function of the m7G in colon adenocarcinoma.

**Methods:**

We established two clusters based on the expression of all genes associated with m7G to explore the expression pattern of 31 key regulatory factors of m7G RNA and assess the prognostic value of regulatory factors. Wilcoxon test and differential box line plots were applied for bioinformatics analysis. Receiver Operating and Kaplan‒Meier curves were utilized to evaluate the prognostic value. Finally, four genes' expression in the colon cancer cell line was confirmed by qRT-PCR.

**Results:**

From The Cancer Genome Atlas database, we found that the expression levels of 25 out of the 31 key N7-methylguanosine RNA modification regulators were significantly different in colon adenocarcinoma. According to 25 methylation regulators’ expression, we identified two subgroups by consensus clustering, in which the prognosis was worse in Group 2 than in Group 1 and was significantly correlated with age. Cluster 2 was significantly enriched in tumor-associated pathways, and immune cells were highly infiltrated in Cluster 1 but weakly infiltrated in Cluster 2. Further results indicated that this risk profile may serve as a standalone predictive factor for colon adenocarcinoma, and the four genetic risk profiles’ prognostic relatedness was successfully verified through Gene Expression Omnibus dataset. At last, A nomogram for prognosis was created according to age, sex, histological grading, clinicopathological staging, and hazard score to accurately predict patient prognosis in colon adenocarcinoma. We successfully validated the differential expression of four genes using qRT-PCR.

**Conclusions:**

In the present study, we revealed the important contribution of key regulators associated with m7G RNA modifications based on all gene expression in colon adenocarcinoma and developed a signature of risk that serves as a promising prognostic marker for patients with colon adenocarcinoma.

**Supplementary Information:**

The online version contains supplementary material available at 10.1186/s12876-023-02657-y.

## Introduction

Global Cancer Statistics 2020 reported that the incidence of colorectal cancer was 10% and that the mortality rate was 9.4%, among all cancers, it is the second most common cause of mortality [1]. Up to 20–30% of colon cancer patients with early-stage illness will develop distant metastases despite complete segmental resection [2]. Colon cancers are not only anatomically different from rectal cancers but also pathologically require different staging procedures. Furthermore, colon and rectal cancers require different neoadjuvant treatments and compatible surgical approaches [3]. The current treatment modalities for colon cancer mainly include surgery and radiotherapy. Although surgery is usually sufficient for early-stage colon cancer, advanced colon cancer requires a combination of preoperative and postoperative radiotherapy [4]. Despite improvements in systemic therapy, the five-year survival rate for CRC metastatic disease patients is around 12–14% [5]. Moreover, patients who are not diagnosed promptly at an early stage often end up with poor treatment outcomes and a poor prognosis. With the development of RNA epigenetics, it has become easier to identify novel biomarkers and therapeutic targets, Moreover, mechanisms of RNA epigenetics may be crucial for improving early cancer diagnosis, treatment, and prognosis.

A regular RNA methylation alteration called m7G has a significant impact on the entire biological process. RNA methylation is a basic process of epigenetic regulation. A large amount of evidence shows that the methylation of RNA plays a crucial function in many biological processes, and RNA methylation’s dysregulation is highly correlated with the development of human cancers, especially gastrointestinal tumors [6]. Ribonucleic acid methylation is a kind of most common RNAs’ posttranscriptional modifications, such as mRNA, miRNA, tRNA, rRNA, snoRNA, and snRNA. The types of RNA methylation are numerous and mainly include N7-methyl guanine, N6-methyl adenosine, 5-methyl cytosine (m5C), 2′-O-methylation, N1-methyl adenosine, pseudouridine, 5-hydroxymethyl cytosine, and adenosine to inosine editing. Moreover, different types of methylated nucleotides are distributed differently and unevenly in different species [7]. RNA methylation participates in many kinds of processes in biology, which included transcription, mRNA translation, circular RNA extensive translation, circulation rhythm, DNA damage responses, heat shock reactions, neurological functions, sex determination, and viral infection [8]. Moreover, gene regulation, DNA repair, and stress responses are also involved in RNA methylation [9, 10]. An mRNA's most common internal modification is N6-methyladenosine (m6A), and studies on m6A have been relatively well-defined and intensive [11]. while m7G (m7G46), located at position 46 of the tRNA nucleotide, is also a kind the most common tRNA modifications [12, 13] and presents in eukaryotes, prokaryotes, and archaea [14], it is relatively poorly studied. 7-Methylguanosine (m7G) can be found in messenger RNA caps as well as defined internal locations in tRNAs and rRNAs [15]. m7G has also been internally detected in human mature miRNAs and pre-miRNAs [15]. m7G may have a significant function in process by which cancer develops. deletion of m7G results in m7G-modified tRNAs as well as an altered cell cycle abundance reduction, which acts as a suppressor of carcinogenesis. Overexpression of METTL1, on the other hand, leads to oncogenic cell transformation and cancer growth [16], including colon cancer [17], lung cancer [11], and intrahepatic cholangiocarcinoma [18]. 7-Methylguanosine is also linked to chemotherapy resistance in tumors, and METTL1 is the most representative enzyme mediating methylation within m7G, which works in concert with its cofactor, WD repeats structural domain 4 (WDR4), to mediate methylation [12].and its mediated 7-methylguanosine (m7G) is essential for the regulation of chemoresistance in cancer therapy [17]. Therefore, N7-methylguanosine (m7G) has a significant role in cancer. Nonetheless, the experimental method of detection is time-consuming, laborious, and costly. Therefore, as a complement to the experimental technique, we developed a prognostic model of colon cancer by all gene expression levels and validated it through the GEO and TCGA databases.

In the present study, transcriptomic data (Source: TCGA (The Cancer Genome Atlas)) dataset were utilized to identify 31 key regulators of colon adenocarcinoma expression. In addition, patients with colon adenocarcinoma were grouped into two clusters according to RNA modification regulator m7G expression patterns by consensus clustering, which used m7G expression status as a criterion, and the two cohorts had significantly different clinical outcomes. Furthermore, based on the infiltration and immune functions of two clusters of immune cells, we compared their diverse characteristics. In addition, we developed a prognostic predictive model of risk signatures, which has good predictive value in patients who suffer from colon adenocarcinoma. As well as that, we successfully validated the Data from the Gene Expression Omnibus (GEO) database on risk signature’s prognostic relevance.

## Materials and methods

### The collection of data

We acquired ribonucleic acid sequencing transcript data and corresponding patients with colon adenocarcinoma information from TCGA about clinical trials (https://portal.gdc.cancer.gov/; through April 8, 2022). In total, 41 normal adjacent tissues and 480 colon adenocarcinoma tissues were included for further analysis. We obtained 31 genes associated with m7G methylation regulation (Table 1). Extraction of expression data for these 31 genes from the TCGA database of the colon adenocarcinoma cohort resulted in expression data for 25 genes for subsequent analysis (Table 2). To enhance reliability, we used 566 colon adenocarcinoma and 19 nontumor samples from the GEO database that contained gene expression data and survival information as an independent cohort (GSE39582) for external validation.

**Table 1:** Related genes regulated by 31 m7G methylation

Abbreviations	Full name
DCP2	Decapping MRNA 2
DCPS	Decapping Enzyme, Scavenger
NUDT1	Nudix Hydrolase 1
NUDT10	Nudix Hydrolase 10
NUDT11	Nudix Hydrolase 11
NUDT16	Nudix Hydrolase 16
NUDT16L1	Nudix Hydrolase 16 Like 1
NUDT3	Nudix Hydrolase 3
NUDT4	Nudix Hydrolase 4
NUDT4B	Nudix Hydrolase 4B
NUDT5	Nudix Hydrolase 5
NUDT7	Nudix Hydrolase 7
AGO2	Argonaute RISC Catalytic Component 2
CYFIP1	Cytoplasmic FMR1 Interacting Protein 1
CYFIP2	Cytoplasmic FMR1 Interacting Protein 2
EIF4E	Eukaryotic Translation Initiation Factor 4E
EIF4E1B	Eukaryotic Translation Initiation Factor 4E Family Member 1B
EIF4E2	Eukaryotic Translation Initiation Factor 4E Family Member 2
EIF4E3	Eukaryotic Translation Initiation Factor 4E Family Member 3
GEMIN5	Gem Nuclear Organelle Associated Protein 5
LARP1	La Ribonucleoprotein 1, Translational Regulator
NCBP1	Nuclear Cap Binding Protein Subunit 1
NCBP2	Nuclear Cap Binding Protein Subunit 2
NCBP3	Nuclear Cap Binding Protein Subunit 3
EIF3D	Eukaryotic Translation Initiation Factor 3 Subunit D
EIF4A1	Eukaryotic Translation Initiation Factor 4A1
EIF4G3	Eukaryotic Translation Initiation Factor 4 Gamma 3
IFIT5	Interferon-Induced Protein With Tetratricopeptide Repeats 5
LSM1	LSM1 Homolog, mRNA Degradation Associated
NCBP2L	Nuclear Cap Binding Protein Subunit 2 Like
SNUPN	Snurportin 1

**Table 2:** Genes obtained by extracting expression data from a colon adenocarcinoma cohort in TCGA database

Name	Expression in tumor tissues
DCPS	High
NUDT1	High
NUDT10	Low
NUDT11	Low
NUDT16	Low
NUDT3	High
NUDT4	High
NUDT5	High
NUDT7	Low
AGO2	High
CYFIP1	Low
EIF4E	High
EIF4E1B	Low
EIF4E3	Low
GEMIN5	High
LARP1	High
NCBP1	High
NCBP2	High
NCBP3	Low
EIF3D	High
EIF4A1	High
EIF4G3	Low
LSM1	High
NCBP2L	Low
SNUPN	High

*TCGA* The Cancer Genome Atlas

### Bioinformatics analysis

#### Identification of m7G RNA modification regulators that are differentially expressed in colon adenocarcinoma

For the detection of differentially expressed m7G RNA methylation regulator genes, we conducted the detection by Wilcoxon test, and a heatmap was created to visualize the results. A false discovery rate (FDR) < 0.05 and an absolute log fold change rate (FC) > 1 were used as significant criteria. Subsequently, a differential box plot was utilized to contrast the expression which is m7G-associated genes among 480 colon adenocarcinomas and 41 regular colon tissues. In order to determine if regulators of m7G RNA methylation are correlated, immune function and infiltration of 480 colon adenocarcinomas and 41 regular colon tissues were displayed by box plot too. Spearman correlation analysis was performed.

#### Consensus clustering dependent on m7G RNA modification regulators was used to separate two cohorts of colon adenocarcinoma patients with varied clinical outcomes

As part of the study, we evaluated the connection between the m7G RNA methylation regulator’s expression and the colon adenocarcinoma’s prognosis. With the ConsensusClusterPlus package in R, two subgroups were created from the cohort of colon cancer patients.. Using the ggplot2 and limma for PCA(Principal Component Analysis), we validated the classification results. Using Kaplan–Meier analysis, a survival curve was plotted for each subgroup for comparing their survivability. Clinical parameters were compared between the two subgroups using a chi-square test. Analysis of Gene Ontology (GO) and Kyoto Encyclopedia of Genes and Genomes [19] (KEGG, https://www.kegg.jp/) data was conducted to functionally annotate the differentially expressed genes in the two subgroups.

#### Development of a prognostic risk model for m7G RNA modification-related genes based on the expression levels of all genes

Univariate Cox regression was used to analyze the associations of m7G-related genes with overall survival. We then performed a minimum absolute shrinkage and selection operator Cox regression (LASSO) to exclude the genes we obtained through the Garment package in R to avoid overfitting. Ultimately, four risk markers associated with m7G-regulated genes based on genome-wide expression were identified. To obtain the risk score, we calculated it obtained by multiplying the gene expression and its coefficients by the least absolute shrinkage and selection operator Cox regression (LASSO) using the following formula: risk score = (0.0859 ∗ HSF4 expression value) + (0.3854 ∗ UPK3B expression value) + (0.0592 ∗ ZNF767P expression value) + (0.1646 ∗ AGAP9 expression value). Patients with colon adenocarcinoma were then divided into low- and high-risk clusters based on the median risk score. The prediction accuracy of the model in predicting prognosis was tested with a receiver operating characteristic (ROC) curve. Chi-square tests were used to evaluate differences between low-risk and high-risk groups in clinicopathological variables. Heatmaps were created to visualize the differences. Differential analysis was also conducted on immune cell infiltration and immune function in the high-risk groups as well as low-risk groups. What’s more, the risk score was also evaluated using multivariate and univariate Cox regression analyses to see if it acted as an independent prognostic indicator.

#### Quantitative reverse transcription-polymerase chain reaction

We used colon cancer cells SW480, and normal intestinal mucosal cells FHC. Total RAN of the cells was extracted using TRIzol method. CDNA was prepared using reverse transcription kit(Novozymes Biotechnology Co., Ltd., Nanjing, China). Quantitative Real-time PCR (qRT-PCR) was performed using SYBER Green I kit and Agilent Technologies Stratagene Mx3000p system(Agilent Co,, Ltd, USA). Finally the results were analyzed using CT values by 2-ΔΔCt. All the primers were purchased from SunYa ( SunY Biotechnology Co., Ltd., Fuzhou, China) and showed in Additional file 1: Table S1.

#### Using GEO's database to validate predictive signatures

For the purpose of validating the four m7G-associated modifier gene risk markers’ predictive value. We used the GSE39582 dataset as a validation cohort. Training cohort patients' scores of risk were determined in the same way we mentioned above. The patients were divided into low-risk as well as high-risk patient clusters by using identical endpoints Kaplan‒Meier survival analysis with ROC curve analysis was then used to evaluate the value of prognosis.

#### Establishment of a prognostic nomogram for adenocarcinoma of the colon

**Fig. 1:**
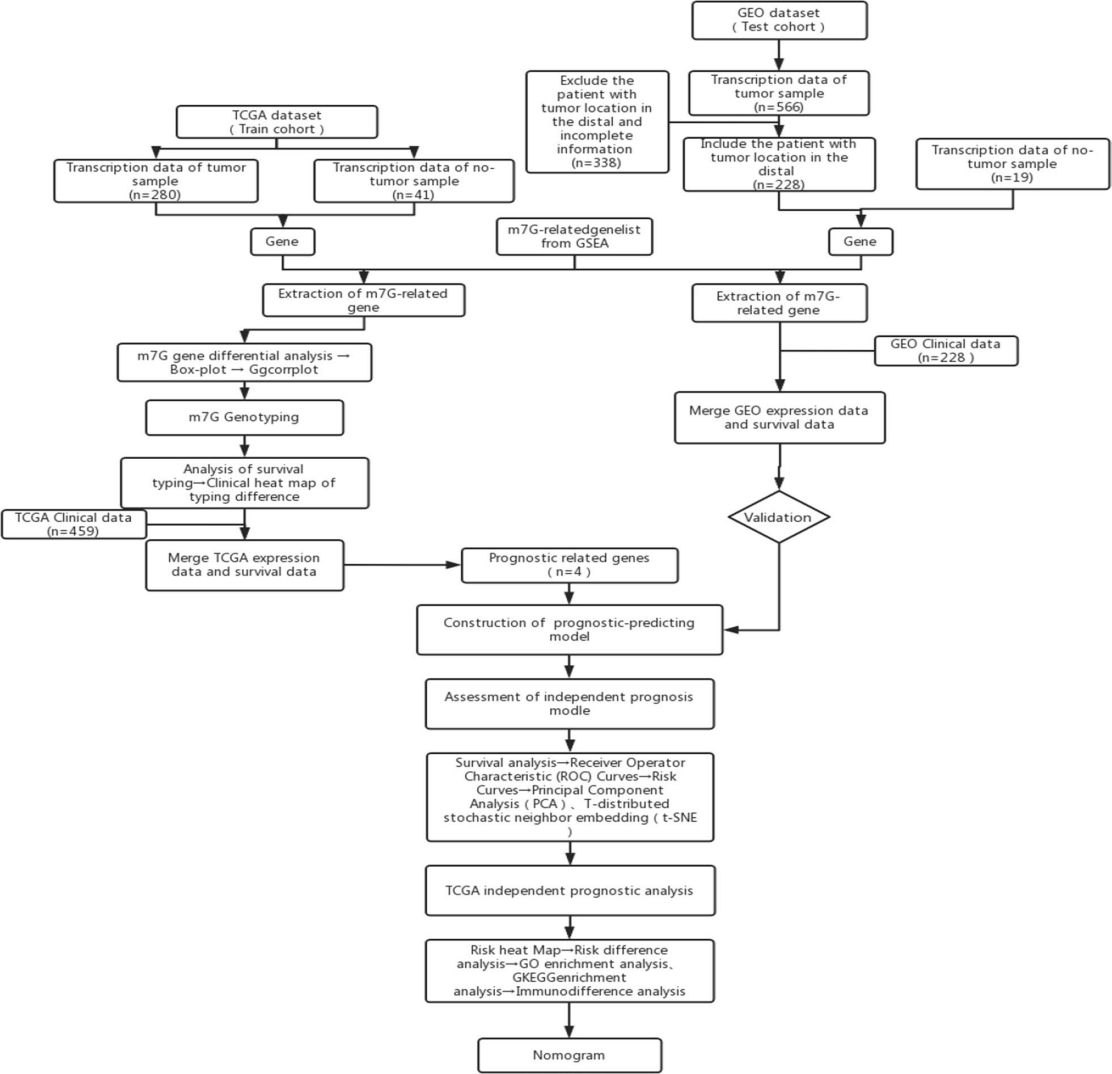
Workflow of the present study. *GEO* Gene Expression Omnibus, *TCGA* The Cancer Genome Atlas, *GO* Gene Ontology, *KEGG* Kyoto Encyclopedia of Genes and Genomes analyses, *GSEA* gene set enrichment analysis

Ultimately, the factors that affect clinical outcomes and the score of risk were used to generate a prognosis nomogram that predicts the patients with colon cancer survival at one, three, and five years by the RMS package. And the clinical factors include sex, age, histological grade, and pathological stage. A workflow of the present study is presented in Fig. 1.

All method descriptions mainly refer to Zhang et al.[20]. All of the abovementioned R packages are available at http://www.Bioconductor.org.

### Statistical analysis

R software (version 4.1.3) was used for all statistical analyses, and a P value of 0.05 was considered as the level of significance.

## Results

### Identification of m7G RNA modification regulators that are differentially expressed in colon adenocarcinoma

**Fig. 2:**
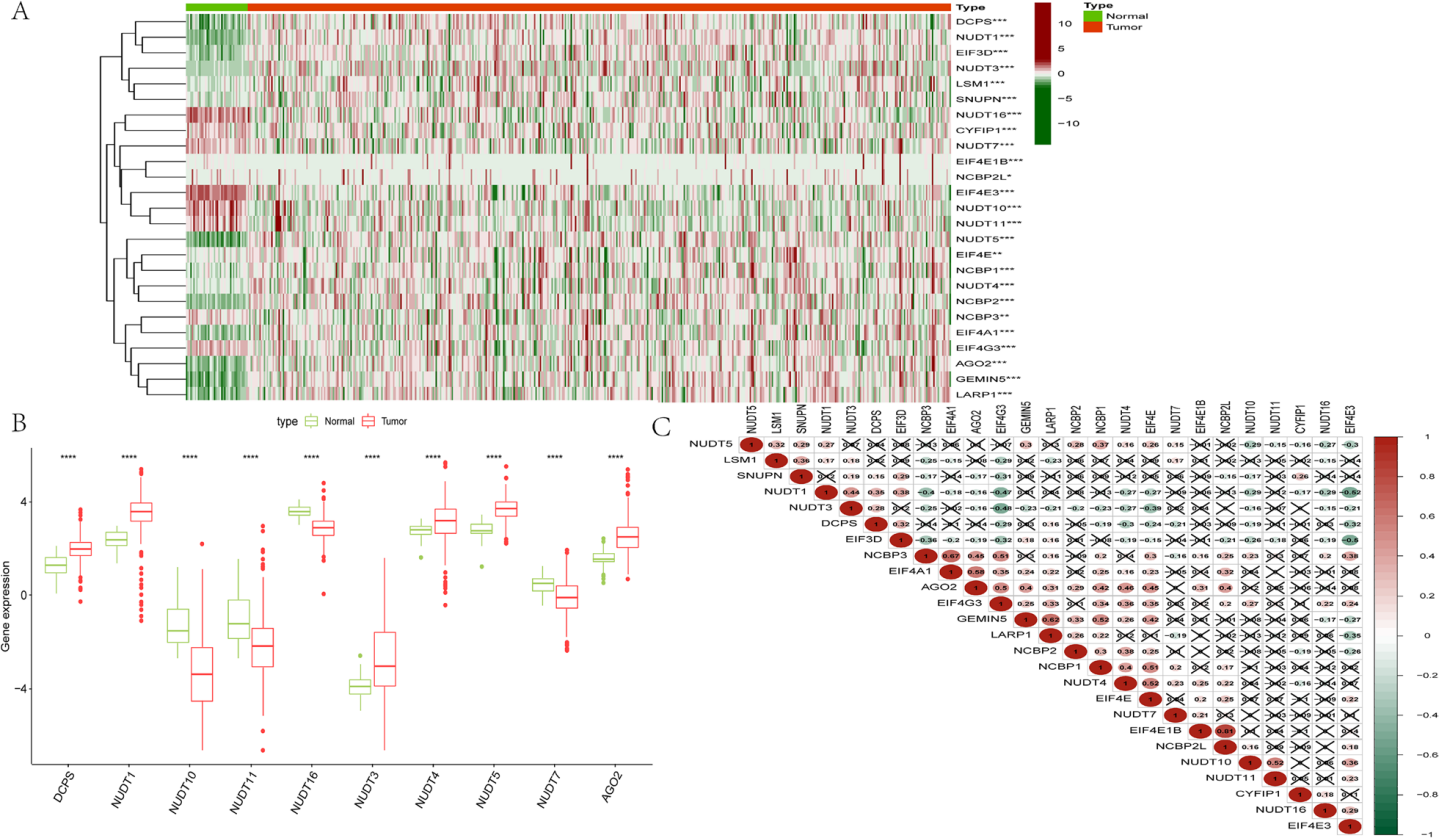
Expression of m7G modification regulators of COAD. **A** Every sample is displayed as a heatmap, showing the expression levels of the m7G RNA modification regulators. Normative samples are indicated by N, while tumor samples are indicated by T. Dark red color and dark green color indicate high and low expression, respectively. The boxplot in **B** shows the multiple COAD m7G RNA modification regulators. Red denotes a COAD sample, while green denotes a normal sample. The interquartile range was 4.40, and there were no outliers. The median value of the expression is shown by the horizontal line in the box. **C** The 25 COAD m7G RNA modification regulators' Spearman correlation analysis. Crosses indicate that there was no association at this time. *p < 0.05; **p < 0.01; ***p < 0.001. COAD, colon adenocarcinoma

It was examined whether 31 genes regulated by m7G are differentially expressed between colon cancers (n = 480) and adjacent tissues (n = 41). Colon cancer tissues expressed differentially most m7G-related genes compared to tissues that are normal as demonstrated by the heatmap (Fig. 2A). We then re-examined gene expression levels in tumors and normal tissues of these 25 differentially expressed genes, and we found that 10 of these genes had significantly different levels of expression. DCPS, NUDT1, NUDT3, NUDT4, NUDT5, and AGO2 had expression levels that were considerably higher in tumor tissues compared in normal tissues (*p* < 0.001), whereas NUDT10, NUDT11, NUDT16, and NUDT7 had expression levels that were considerably lower in tumor tissues compared in normal tissues (*p* < 0.001) (Fig. 2B). To understand the intrinsic relationship among the 25 m7G RNA modification regulators, an analysis of correlation was conducted. According to Fig. 2C, it appears that EIF4E1B and NCBP2L have the strongest association A negative correlation was found between EIF4E3 expression and NUDT1, while EIF4E1B expression, as well as NCBP2L, showed a positive connection.

### Consensus clustering dependent on m7G RNA modification regulators was used to separate two cohorts of colon adenocarcinoma patients with varied clinical outcomes

**Fig. 3:**
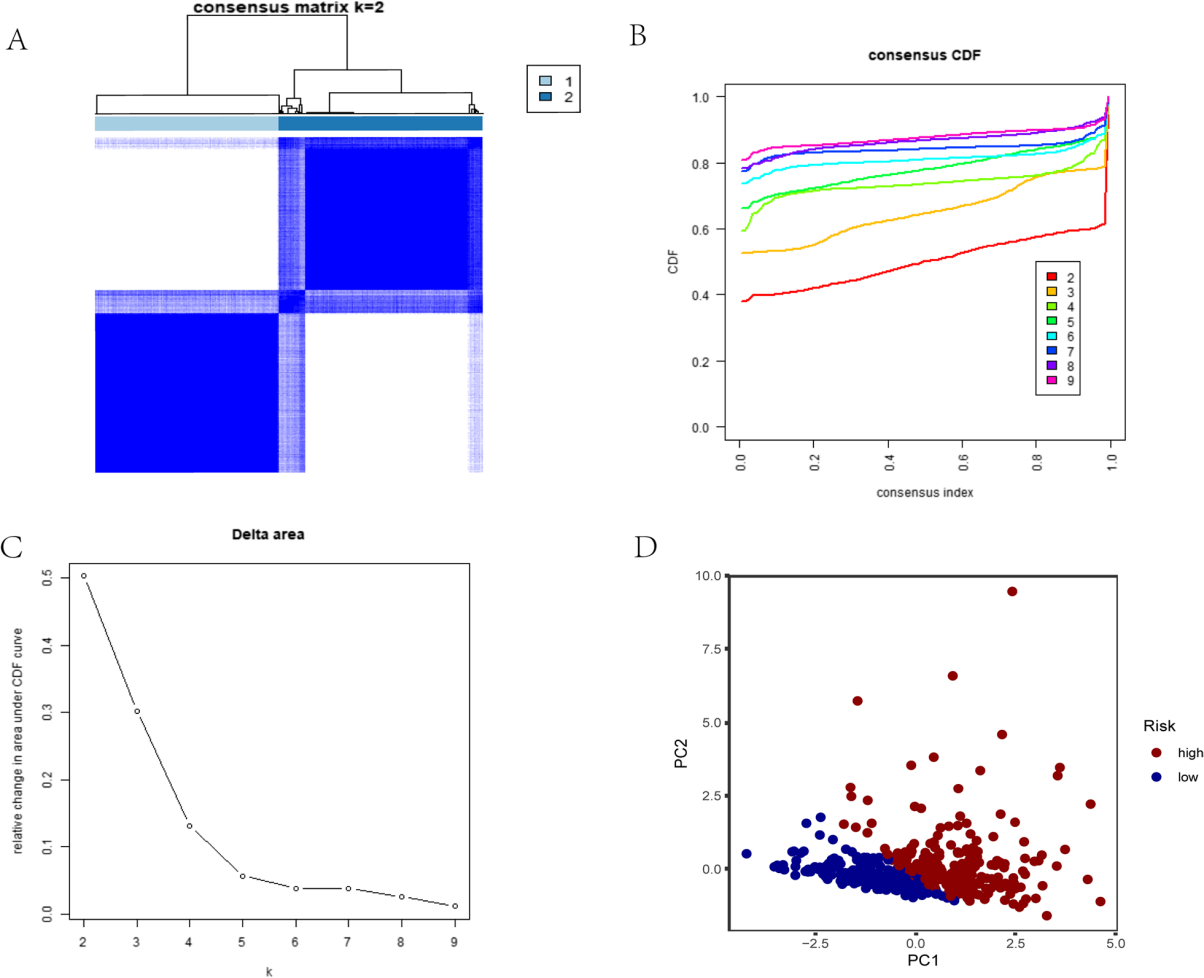
Consensus cluster analysis of COAD. **A** Subgroup correlation when using the k = 2 clustering factor. **B** For k = 2–9, the cumulative distribution function (CDF) is shown in the picture. **C** In the CDF curve for k = 2–9, the corresponding variance is in the area below the CDF curve. **D** An analysis of RNA-seq data based on principal components. Blue dots indicate low-risk clusters, whereas red dots indicate high-risk clusters

**Fig. 4:**
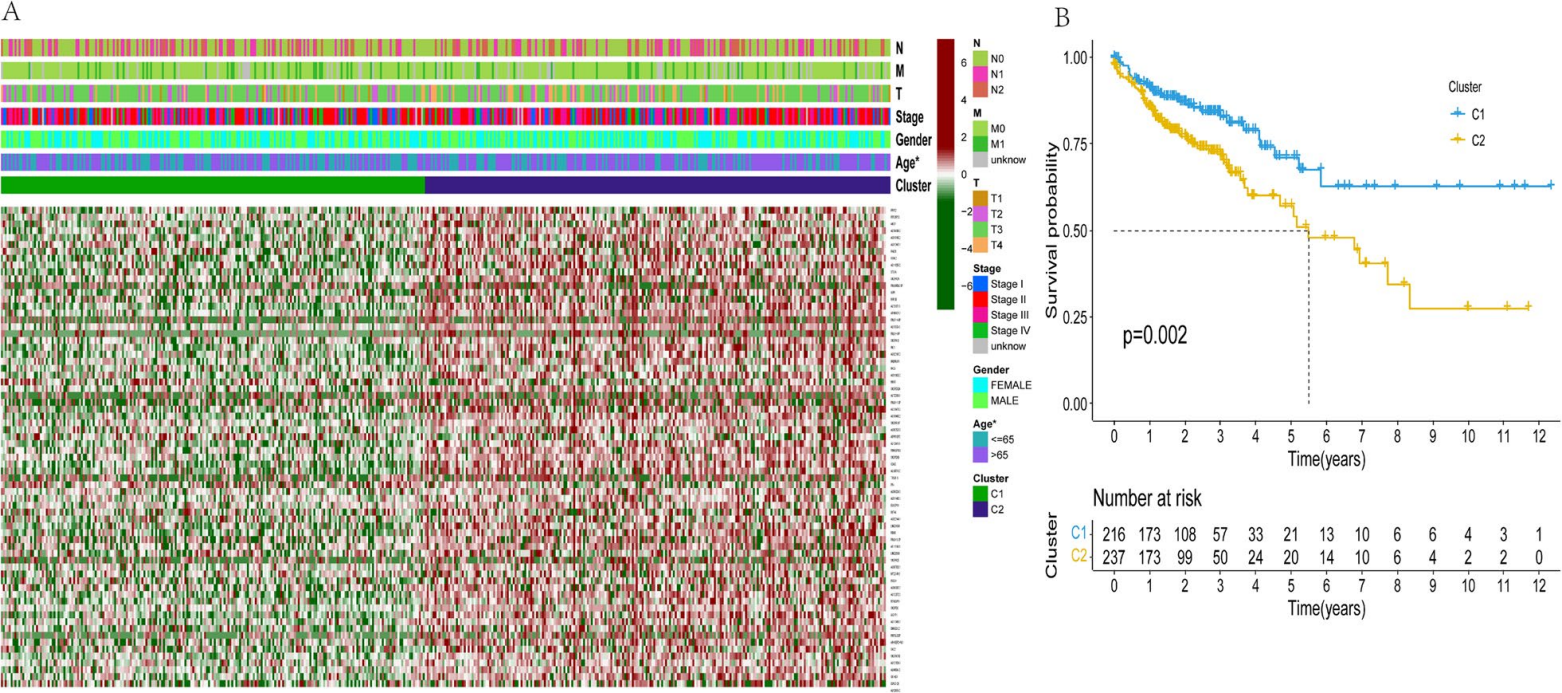
Differences in clinical and pathological characteristics of Clusters 1 and 2 as well as overall survival. **A** Here is a heatmap showing the clinicopathological characteristics of these two clusters. Red and green represent high and low expression, respectively. The differences in age were significant (*p* < 0.05). **B** Among Clusters 1 and 2, an OS Comparison is made. *p < 0.05; **p < 0.01; ***p < 0.001. OS, overall survival

To further explore clinical importance of the 25 m7G RNA modification regulators, and by analyzing cancer-related gene expression profiles, we divided them into groups. By using m7G RNA modification regulators that are similar, a clustering technique with k = 2 was able to divide a colon cancer cohort into two discrete and nonoverlapping clusters. (Fig. 3A–C). We used principle component analysis (PCA) to further investigate the two classes for the purpose of verifying clustering results. Significant differences between Clusters 1 and 2 were visible in PCA plots (Fig. 3D). We next evaluated whether there were notable variations in clinical parameters and overall survival (OS) between these two groups. As a consequence, OS in cluster 1 was considerably superior to OS in cluster 2 (*p* < 0.01) (Fig. 4B). Furthermore, Cluster 2 showed upregulated expression levels of most RNA modification regulator genes rather than Cluster 1 (Fig. 4A). Although there were no significant variations in histological grading, pathological stage, or sex, age variances between the two groupings were noticeably different. (*p* < 0.05) (Fig. 4A). Consensus clustering's findings, therefore, showed a strong correlation between colon adenocarcinoma's malignancy and patterns of expression of RNA modification regulators in m7G.

**Fig. 5:**
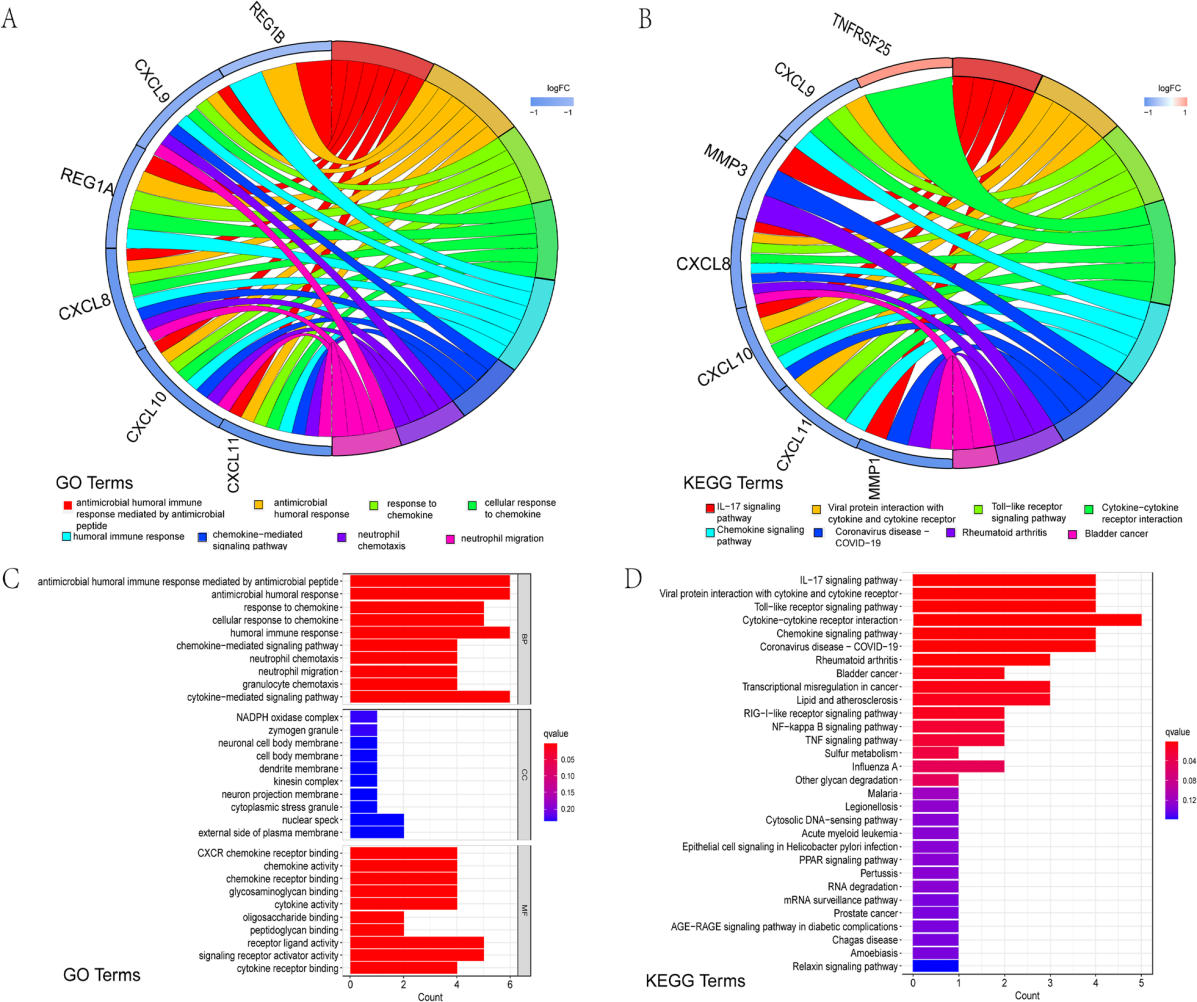
Genes with differential expression between two clusters as determined by GO and KEGG studies. According to GO (**A**, **B**) and KEGG pathway analyses, more genes in Cluster 2 were functionally annotated (**C**, **D**). *GO* Gene Ontology, *KEGG* Kyoto Encyclopedia of Genes and Genomes

We next performed GO and KEGG analyses of the differentially expressed genes between Clusters 1 and 2 to further explain the outcomes of clustering in terms of underlying biological processes. According to analysis of GO, genes which are downregulated were mainly involved in biological processes connected to cancer listed below: neutrophil chemotaxis and migration; antimicrobial humoral immune response mediated by an antimicrobial peptide; antimicrobial humoral response; response to chemokine; cellular response to chemokine and humoral immune response; and response to chemokine (Fig. 5A, B). In addition, according to KEGG analysis, the upregulated genes are associated with cytokine-cytokine receptor interactions, and the majority of the downregulated genes were associated with COVID-19, a coronavirus illness (Fig. 5C, D).

### Development of a prognostic risk model for m7G RNA modification-related genes based on the expression levels of all genes

**Fig. 6:**
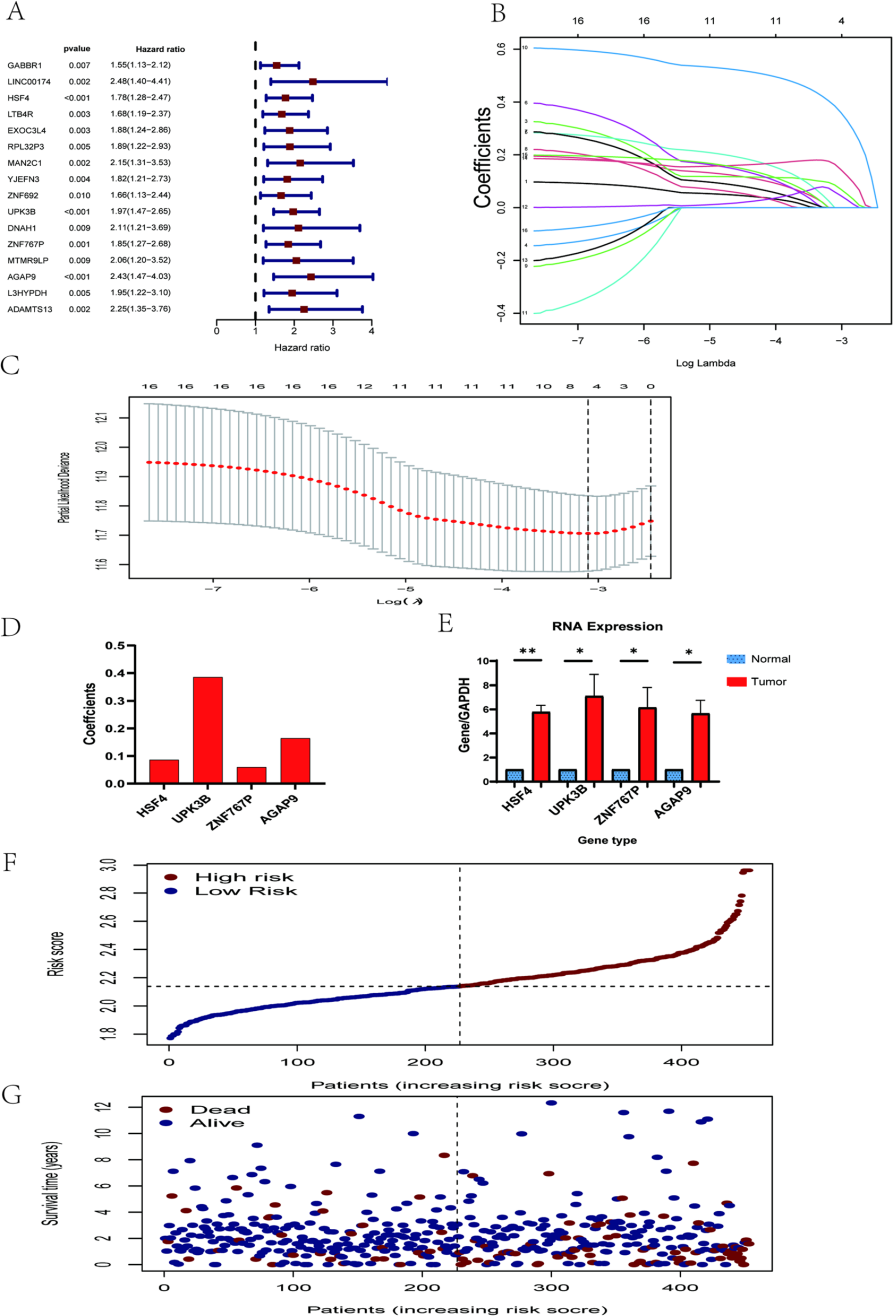
Establishment of a model for predictive disease risk based on the regulator genes of RNA modification genes in m7G. **A** Analysis of the regulator genes correlated with m7G RNA methylation using univariate Cox regression. **B**–**D** The procedure utilized to create the signature with Cox regression and the absolute shrinkage and selection operator (LASSO). **E** Expression levels of the four genes in FHC (normal) and SW480 (tumor) cells. **F** Risk score distributions of the risk score model. **G** A prognostic model of survival status distributions. *p < 0.05; **p < 0.01; ***p < 0.001

Our study performed univariate Cox regression in order to determine whether expression levels of the important regulators are connected to a patient's prognosis for colon cancer. The findings demostrated that OS as well as 16 of these genes existed significant conection (*p* < 0.01) (Fig. 6A). Among these 16 regulators, GABBR1, LINC00174, HSF4, LTB4R, EXOC3L4, RPL32P3, MAN2C1, YJEFN3, ZNF692, UPK3B, DNAH1, ZNF767P, MTMR9LP, AGAP9, L3HYPDH, and ADAMTS13 were considered risk genes with HR (hazard ratio) > 1. Among them, LINC00174, MAN2C1, DNAH1, MTMR9LP, AGAP9, and ADAMTS13 were at higher risk with HR > 2. Subsequently, all gene-modifying regulators with the highest prognostic ability were screened by LASSO Cox regression analysis (Fig. 6B, C), which identified the following four genes to estimate the risk of colon adenocarcinoma: heat shock transcription factor 4 (HSF4); uroplakin 3B (UPK3B); zinc finger family member 767, pseudogene (ZNF767P); and ArfGAP with GTPase domain, ankyrin repeat and PH domain 9 (AGAP9) (Fig. 6D). Calculation of risk score in following formula: risk score = (0.0859 HSF4 expression value) + (0.3854 UPK3B expression value) + (0.0592 ZNF767P expression value) + (0.1646 AGAP9 expression value). The four genes highly expressed in tumor cells were verified by qRT-PCR (Fig. 6E). Additionally, Fig. 6F shows the risk distribution score of patients with colon adenocarcinomas, and each patient's survival status was shown using a scatter plot (dot plot) (Fig. 6G).

**Fig. 7:**
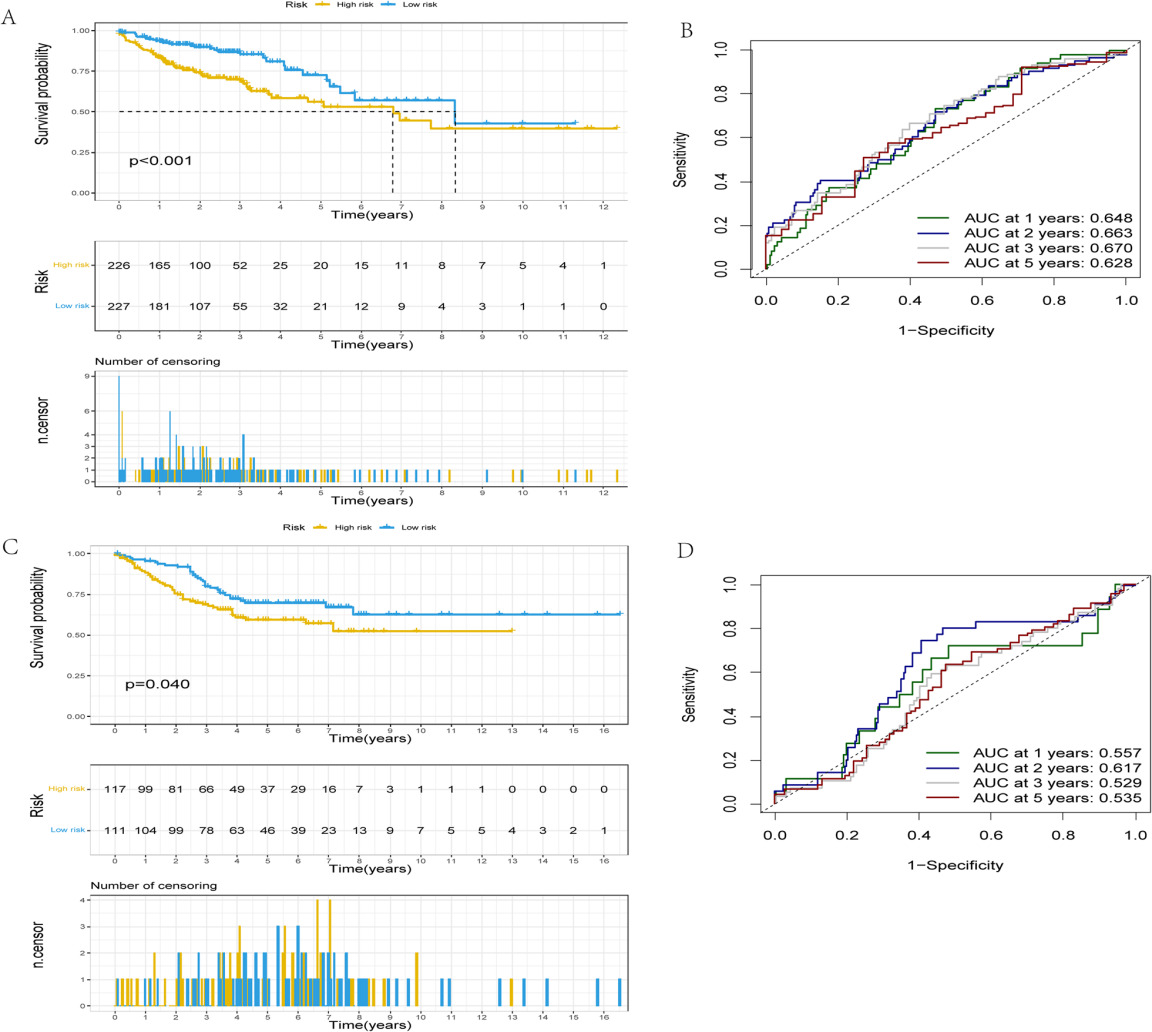
Prognostic model survival analyses based on Kaplan–Meier curves. Using the median risk score as the cutoff, patients from the two datasets were divided into low-risk (blue) and high-risk (yellow) groups. **A** In TCGA cohort, the group which is in low risk had a higher probability of survival compared to the group which is in high risk (*p* < 0.001). **B** The one-, two-, three- and five-year AUC values were 0.648, 0.663, 0.670, and 0.628, in the TCGA cohort. **C** In GEO cohort, the model for prognosis was verified to be accurate. The low-risk group's chances of survival were higher than those of the high-risk group (*p* = 0.04). **D** with one-, two-, three-, and five-year AUC values of 0.557, 0.617, 0.529, and 0.535, respectively. *GEO* Gene Expression Omnibus, *TCGA* The Cancer Genome Atlas, *AUC* area under the curve

In line with median risk scores, colon cancer patients were divided into low and high risk groups to investigate the four-gene signature model's prognostic effects. According to the survival analysis, there was a worse overall survival rate (OS) for patients with high-risk scores than for those with low-risk scores (Fig. 7A, *p* < 0.001). In the high-risk group, the five-year OS rate was 56.3%, while in the low-risk cluster, the five-year OS rate was 73.3%. The area under the curve (AUC) values for the one-year, two-year, three-year, and five-year OS were 0.648, 0.663, 0.670, and 0.628, respectively, as determined by ROC curve analysis, which indicated the strong predictive potential for survival outcomes (Fig. 7B).

### Using GEO's database to validate predictive signatures

GEO's database microarray data (GSE39582) as a testing set were conducted to evaluate the four-gene signature's prognostic value. According to the cut-off values of the TCGA cohort, 228 individuals with colon adenocarcinoma in the GSE39582 cohort were grouped into two groups. 117 people were in the category of having high risk. About another 111 people were defined as low risk. Survival analysis showed that patients with colon cancer in the low-risk group had a considerably better OS than patients in the high-risk group, which is in line with the findings in the TCGA cohort. (Fig. 7C , *p* = 0.04). In the one-, two-, three-, and five-year OS, the AUC values were 0.557, 0.617, and 0.535., which demonstrated that the prediction model accurately predicted the OS of colon adenocarcinoma patients (Fig. 7D).

### Prediction of colon cancer patient prognosis using the four-gene risk signature

**Fig. 8:**
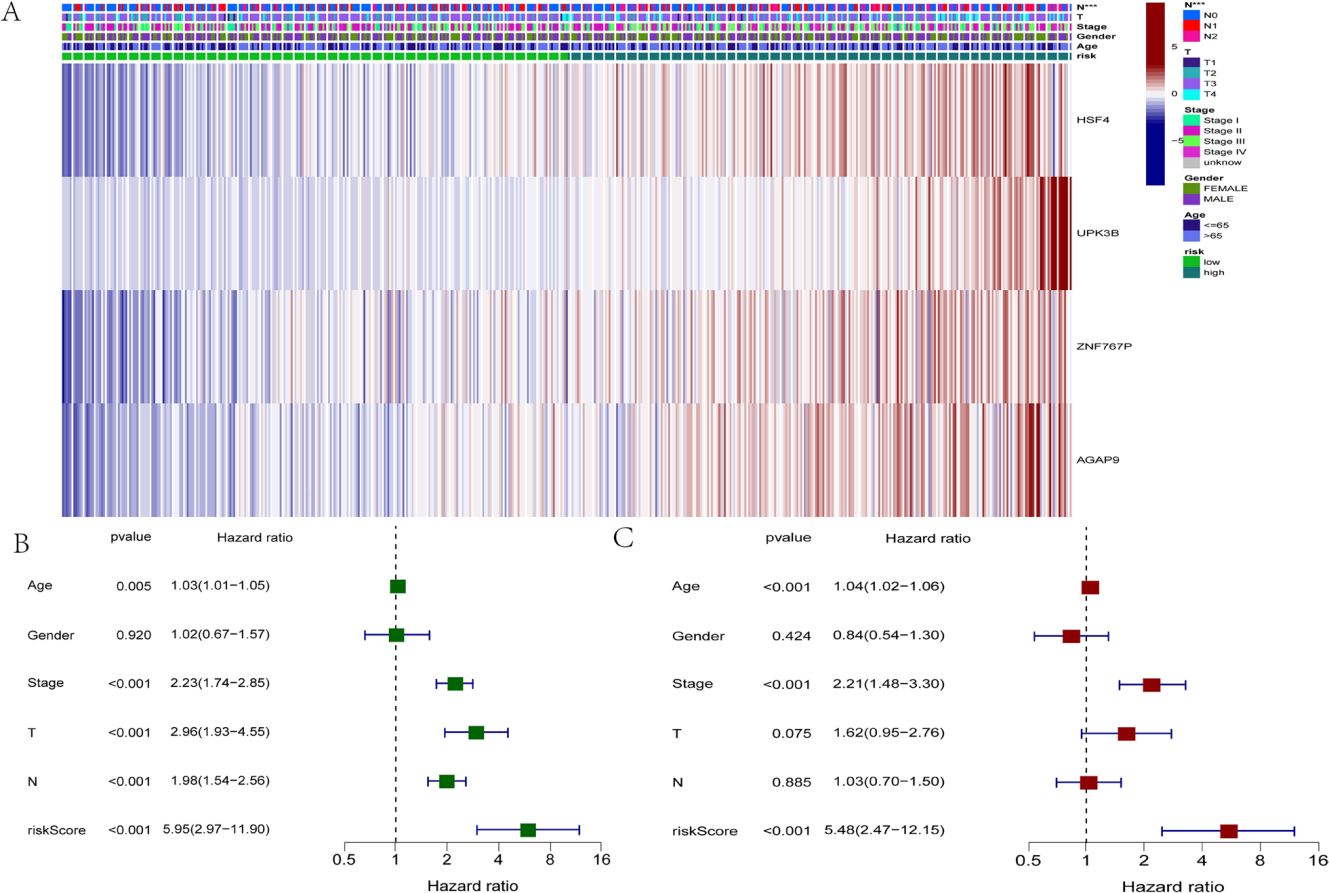
The prediction ability of the risk score and clinicopathological characteristics for COAD patient prognosis. **A** The heatmap displays the expression of five m7G RNA modification regulators and the distribution of clinicopathological characteristics in high- and low-risk groups. In the heatmap, it displays the expression levels of five m7G RNA modification regulators, as well as the clinicopathological characteristics’ distribution in groups at high- and low-risk. **B** Clinicopathological parameter and OS assessments using univariate Cox regression. **C** Clinicopathological variables and OS were analyzed using multivariate Cox regression. *p < 0.05; **p < 0.01; ***p < 0.001. *COAD* colon adenocarcinoma, *OS* overall survival

Comparing low and high risk groups based on pathological stage N revealed significant differences in clinical parameters (*p* < 0.001). The heatmap shows the expression and clinical correlation of four genes, and all four genes are strongly associated with prognosis between the two groups. (Fig. 8A). In total, 458 cases were added to the Cox regression analysis after cases with insufficient clinical information were eliminated. According to univariate analysis, OS is significantly associated with four-gene risk scores, T stage, N stage, and clinicopathological stage in patients with colon adenocarcinoma (Fig. 8B , *p* < 0.001). In order to ascertain if four-gene risk marker is a predictive marker for colon adenocarcinoma independent of the other clinicopathological characteristics, multivariate Cox regression analysis was performed. The results revealed that OS in individuals with colon adenocarcinoma was independently correlated with risk score and clinicopathological stage. (Fig. 8C , *p* < 0.001). These findings indicated that the four-gene risk signature can be utilized as an indicator which can independently prognosticate for colon adenocarcinoma regardless of sex, age, histological grade, and pathological stage.

### Differential analysis of immune cell infiltration and immune function in groups at high- and low-risk

**Fig. 9:**
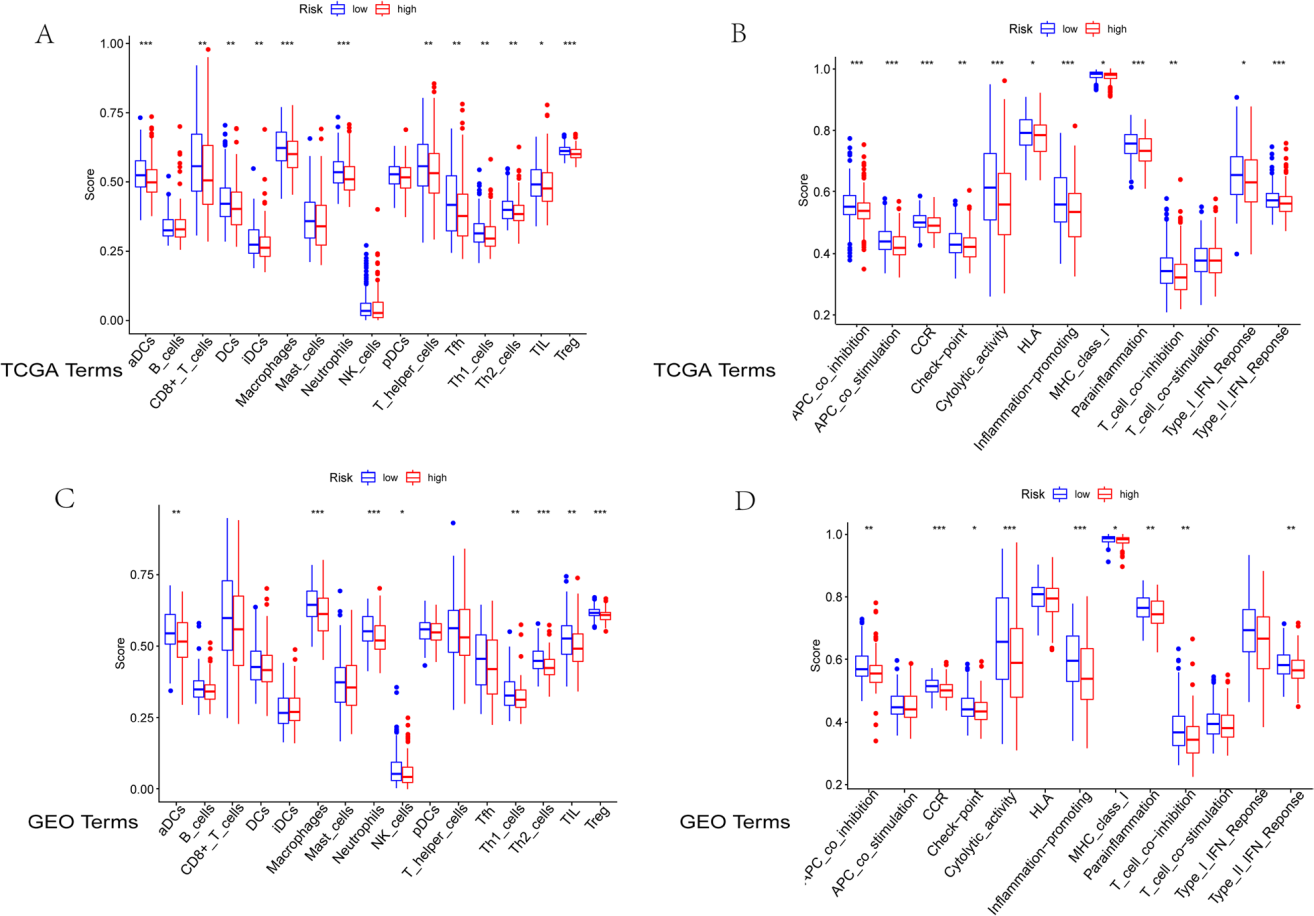
Analysis of immunological differences between high- and low-risk groups of COAD patients. Groups at low risk are represented by the blue box, while groups at high risk are represented by the red box. The horizontal line in the box showed the median value, which is the expression of different groups. **A** The levels of macrophages, neutrophils, and regulatory T cells (Tregs) were high in the group at low risk but low in the group at high risk (*p* < 0.001). **B** In contrast to the group at high risk, the levels of APC coinhibition, APC costimulation, and CCR were low in the group at low risk (*p* < 0.001). **C** The levels of macrophages, neutrophils, and Tregs were significantly higher in the group at low risk and significantly lower in the group at high risk (*p* < 0.001). **D** The levels of CCRs, cytolytic activity, and inflammation promotion were high in the group at low risk but low in the group at high risk. *p < 0.05; **p < 0.01; ***p < 0.001. COAD, colon adenocarcinoma; CCRs, chemokine receptors

We examined the variations in immune cell infiltration connected to colon cancer in the TCGA database. Macrophage, neutrophil, and regulatory T cells (Tregs) infiltration levels were high in group which is at low risk but low in the group which is at high risk (*p* < 0.001) (Fig. 9A). Differences in immune functions associated with colon adenocarcinoma were analyzed. The levels of APC coinhibition, APC costimulation, chemokine receptors (CCRs), cytolytic activity, inflammation promotion, parainflammation, and type-II IFN-response were high in the low-risk group but low in the high-risk group (*p* < 0.001) (Fig. 9B).

Immune cell differential analysis was also verified to be significant in the GEO database with high infiltration levels of macrophages, neutrophils, and Tregs in the group which is at low risk but low infiltration of these cells in the group which is at high risk(*p* < 0.001) (Fig. 9C). Regarding immune function differential analysis, the levels of CCRs, cytolytic activity, and inflammation promotion were high in the group at low risk but low in the group at high risk (*p* < 0.001) (Fig. 9D).

### Construction of a nomogram for colon adenocarcinoma prognosis

**Fig. 10:**
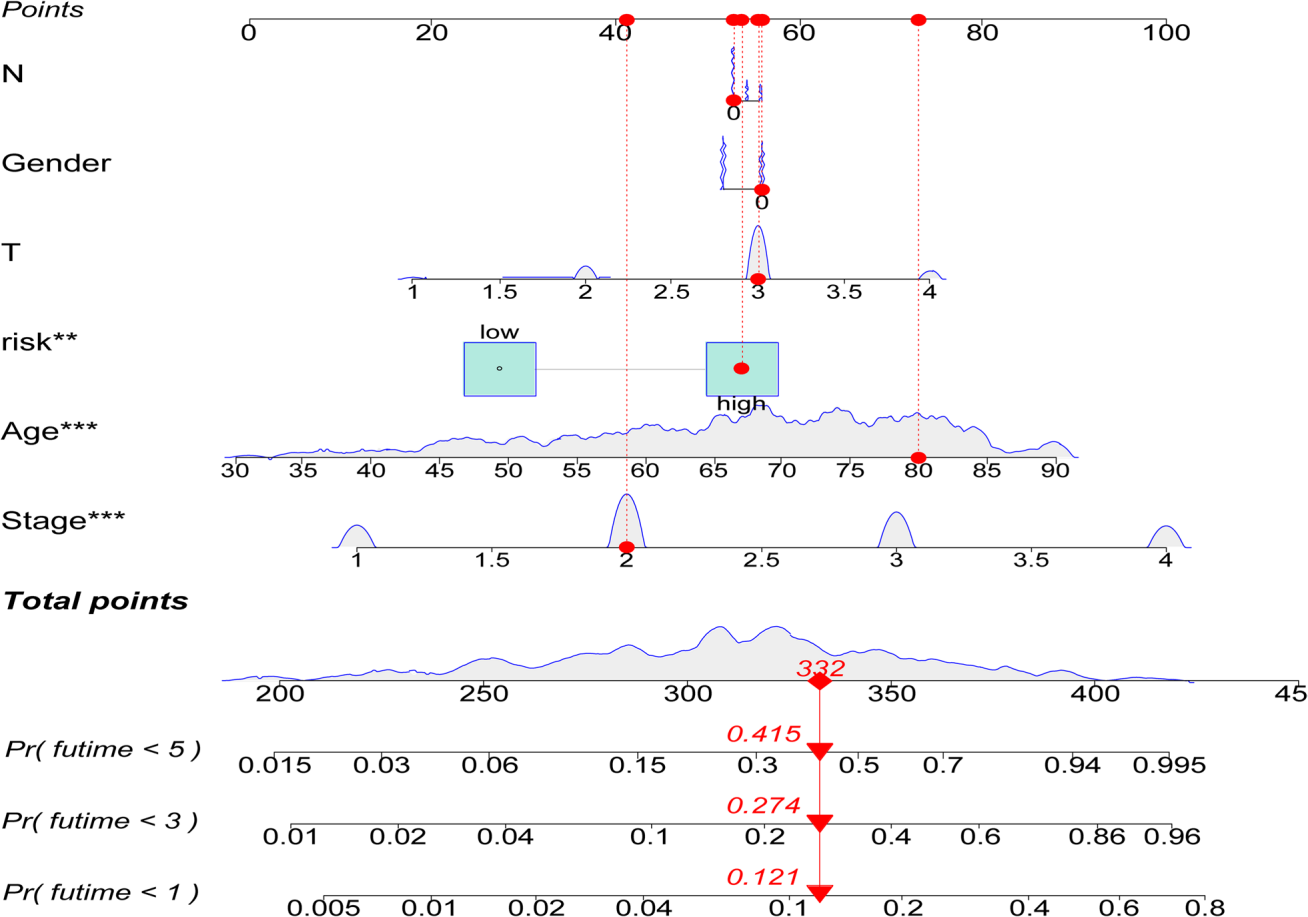
Establishment of a predictive nomogram using a risk score and clinicopathologic traits

To establish a quantitative method to predict individual survival, we created a novel predictive nomogram based upon age, sex, histological grade, pathological stage, and risk score. (Fig. 10). The outcomes demonstrated that in patients with colon adenocarcinoma, one, three, and five-year OS were systematically predicted by the nomogram.

## Discussion

Based on four modifiers of key regulators closely correlated with prognosis in patients with colon adenocarcinoma, a predictive model was constructed by us. Patients with colon adenocarcinoma can have their prognosis reliably predicted using the established risk score. After selecting patient data from TCGA database that satisfied the inclusion requirements, we first performed a bioinformatics analysis. To find genes that were differently expressed, we performed the Wilcoxon test in R. And these genes can encode regulators of m7G RNA methylation. In colon adenocarcinoma, we then identified the differentially expressed m7G RNA modification regulators. Using the screened gene expression patterns in accordance with k = 2, we divided the colon cancer patients into two groups with distinct clinical outcomes. We also analyzed the variations in immune function and immune cell infiltration between the two groups. Following these procedures, we created the predictive risk model which is based upon all levels of gene expression. Finally, to validate the prognostic model, we repeated the above steps to validate the prognostic model by screening the data of colon adenocarcinoma patients who met the inclusion criteria through the GEO database, which was well validated. Based on the accuracy of the results, we evaluated the prognosis of colon adenocarcinoma patients by generating a nomogram.

Heat shock transcription factor 4 (HSF4) is a heat shock factor and is a member of the HSF family. HSF4 has various physiological functions as follows: regulating the transcriptional program of the heat response or stress response; regulating cell proliferation and differentiation during development; regulating DNA damage repair, and regulating normal physiological processes. HSF4’s Alterations are also strongly linked to cataracts, cancer, and other illnesses [21]. Mice lacking HSF4 produce irregular lenses and develop cataracts early in experiments [22–24]. A previous study on a cohort of patients with congenital cataracts in China has indicated that disease development is closely associated with genetic mutations in HSF4 DBD [25]. By playing a critical role in carcinogenesis and tumor progression, HSF4 has been demonstrated to enhance EMT by activating the AKT pathway in a HIF1α-dependent manner in hepatocellular carcinoma; Hepatocellular carcinoma cells are better able to migrate, disseminate, and invade when HSF4 is upregulated, which promotes aggressive tumor behavior, indicating that high HSF4 expression may be a predictor of poor hepatocellular carcinoma after radical resection [26].

The expression of uroplakin 3B (UPK3B), Several tissues, and organs have been shown to include a few of the main structural elements of uroepithelial tissue (UPK3A and UPK3B). For example, in mouse embryos, Cre recombinase activity driven by UPK3B is detected in the liver, heart, kidney, lung, and neural crest cells. UPK3B expression has been detected in mouse testes, epididymal spermatozoa, ovarian follicles, and oviductal mucosa, proving that UPK3B may be extremely important for the development of mouse gametes as well as gamete delivery organs [27]. A whole transcriptome analysis of placental changes in fetuses with prenatal arsenic exposure has reported that UPK3B is one of the most significant ‘off’ genes for arsenic exposure in females [28]. Transcriptome analysis of liver fibrosis has identified UPK3B as a potential regulator of hepatic stellate cell (HSC) activation-induced liver fibrosis [29]. Low FOXA1 expression has been linked to earlier tumor staging, while FOXA1 deletion has been associated with high histological grade. Increased UPK3B expression, decreased E-calcineurin expression, and increased cell proliferation have been observed in FOXA1-deficient RT4 bladder cancer cells, demonstrating a strong relationship between high UPK3B expression and tumor malignancy [30].

These recent studies have demonstrated that HSF4 and UPK3B are both closely associated with tumors. Although few studies have been reported on the ZNF767P and AGAP9 genes, they are promising research targets. What’s more, by analyzing immune cell infiltration and immune activity differently, macrophages contribute a significant part in the formation of tumors too. while the therapeutic effect can also be achieved through the modulatory role of engineered macrophages in the tumor immune microenvironment and tumor therapy [31]. A dysregulation of macrophage-mediated immunosuppression leads to chronic inflammation at low grade due to tissue-specific macrophages and neutrophils, which ultimately leads to the development of cancer [32]. Neutrophils are considered complex cells with many specific functions, and they act as effectors of the innate immune response and play a regulatory role in multiple processes, such as cancer, acute injury, repair, autoimmunity, and chronic inflammation [33]. In the host, neutrophils reflect inflammation, which is a hallmark of cancer [34]. An association between high layilin (LAYN) expression and poor overall survival in colon cancer patients has been demonstrated by Pan et al.. A positive correlation exists between LAYN expression and macrophage and neutrophil infiltration in colon adenocarcinoma (COAD) [35]. The immune system tightly controls Th17/Treg homeostasis through the TGF-/IL-2 and IL-6 cytokine axis. Regulatory T cells (Tregs) are necessary for self-tolerance and defense against autoimmunity, and they are typically linked to the advancement of cancer [36]. By maintaining Treg activity and accumulation in the colon, glycoprotein-A repetition predominant (GARP) reduces cancer immunity [37]. Thus, these findings indicate that macrophages, neutrophils, and Tregs are highly infiltrated in the group at low risk, which reduces the tumorigenesis development.

According to the current study, three immune function differential analyses were validated in both TCGA and GEO databases. Numerous elements of cancer biology have been identified to involve chemokines and their receptors; their possible targets have been evaluated in several studies, and chemokine receptor inhibitors have been used in clinical practice in hematologic malignancies [38]. In patients with gastric cancer, the cytolytic activity score can be employed as a biomarker in antitumor immunity and clinical prognosis [39] but also to evaluate anticancer immunity in colorectal cancer [40]. Nuclear factor-κB (NF-κB), which promotes inflammation, is a central mediator of the inflammatory process, and activation of NF-κB is also prevalent in cancer, which is mainly driven by inflammatory cytokines in the tumor microenvironment [41]; thus, inflammation promotion plays a crucial role in tumorigenesis. In conclusion, chemokine receptors, cytolytic activity, and inflammation promotion are closely related to tumors and play critical roles in the diagnosis, treatment, and prognosis of tumors.

The present study had several benefits. First, based on the patterns of major regulators related to m7G RNA alterations expressed in all genes, we generated the first predictive model for colon cancer. Second, the model was constructed using a variety of statistical techniques, and both the test cohort and the entire cohort were used for validation. As a result, the predictive risk model for patients with colon cancer is precise and trustworthy. The accuracy of the risk score model in predicting OS was higher than that of pathological stage and age, and the risk score model can be employed as a standalone prognostic indicator. Finally, throughout the advancement of colon cancer research, our model can also be utilized to predict immune cell infiltration and to study differential immune function. However, the present study had several limitations. First, we generated an unvalidated prognostic risk model based on a public database rather than a clinical study. In addition, the possible mechanisms through which the important regulators of m7G RNA modification affect colon cancer progression need to be further investigated by basic experiments. Further, there was a lack of in vivo or in vitro experiments exploring the molecular functions of the four genes in the model. Further studies are required to elucidate the mechanism.

## Conclusions

In the present study, a four-gene signature of colon cancer, consisting of HSF4, UPK3B, ZNF767P, and AGAP9, was generated and validated. It can be used as an auxiliary predictive variable, and patients with colon adenocarcinoma can be predicted how long their survival will last using this analysis.

## Supplementary Information

**Additional file 1.** The original code and visual data.

## Data Availability

All datasets presented in this study are included in the article/supplementary material. All the R packages above are available from http: //www.Bioconductor.org.

## Abbreviations

m7G: N7-methylguanosine
COAD: Colon adenocarcinoma
ROC: Receiver operating characteristic curves
TCGA: The Cancer Genome Atlas
GEO: Gene Expression Omnibus
FC: Fold change rate
FDR: False discovery rate
KEGG: Kyoto Encyclopedia of Genes and Genomes
GO: Gene ontology
LASSO: Selection operator Cox regression
GSEA: Gene set enrichment analysis
PCA: Principal component analysis
OS: Overall survival
CDF: The cumulative distribution function
HR: Hazard ratio
AUC: Area under curve
