# Supplementary material for: Expression patterns and prognostic value of key regulators associated with m7G RNA modification based on all gene expression in colon adenocarcinoma
Source: BMC Gastroenterol. 2023 Jan 21;23:22. doi: 10.1186/s12876-023-02657-y (PMC9867544; doi:10.1186/s12876-023-02657-y)
Supplement: Supplementary file 1 — Additional file 1. The original code and visual data. [file 12876_2023_2657_MOESM1_ESM.zip › 12876_2023_2657_MOESM1_ESM/10. Clinical heat map of fractional differences.zip!/10. Clinical heat map of fractional differences/heatmap.pdf]

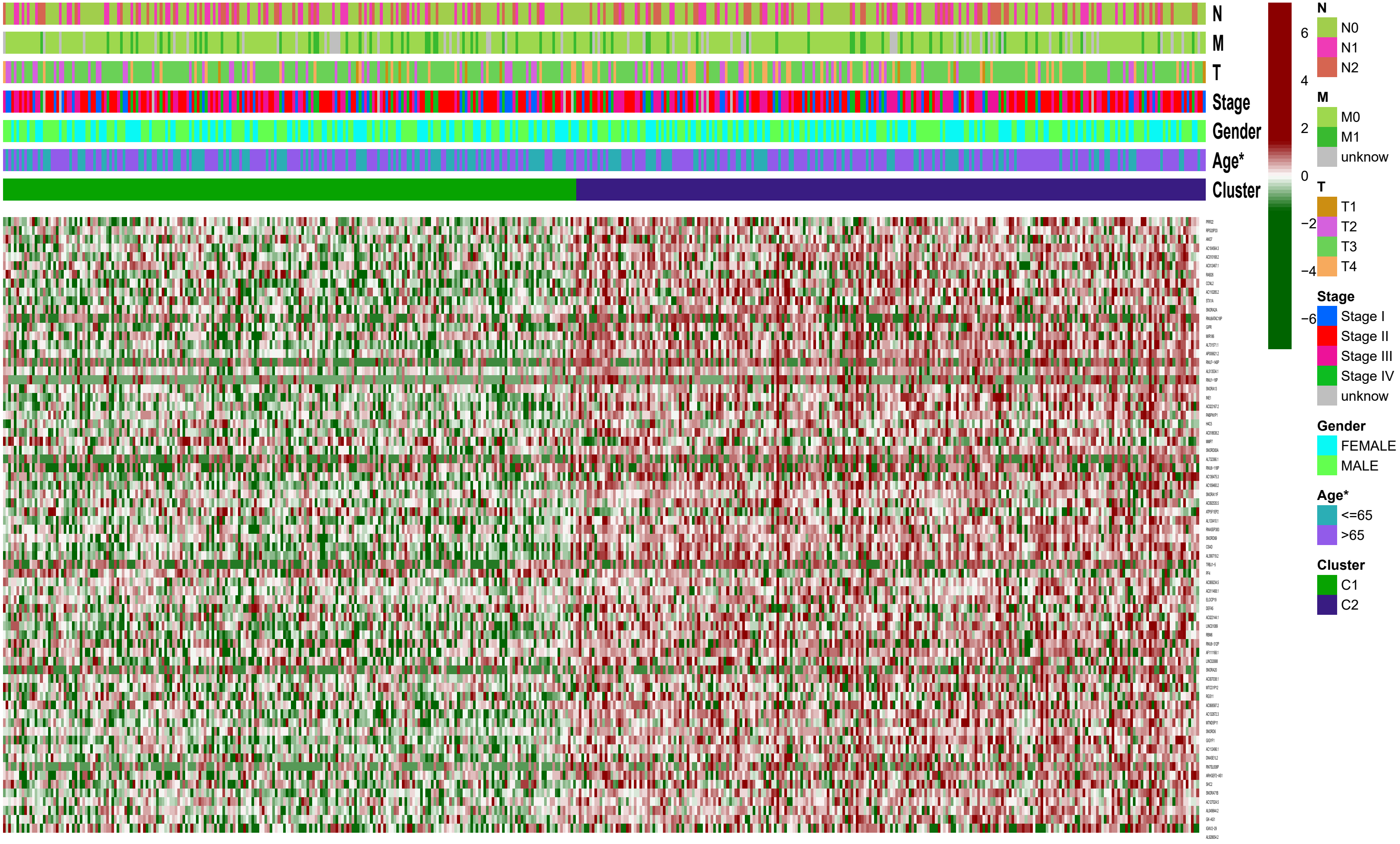
N
M
T
Stage
Gender
Age*
Cluster
6
4
2
0
−2
−4
−6
N
N0
N1
N2
M
M0
M1
unknow
T
T1
T2
T3
T4
Stage
Stage I
Stage II
Stage III
Stage IV
unknow
Gender
FEMALE
MALE
Age*
<=65
>65
Cluster
C1
C2
