# Supplementary material for: Expression patterns and prognostic value of key regulators associated with m7G RNA modification based on all gene expression in colon adenocarcinoma
Source: BMC Gastroenterol. 2023 Jan 21;23:22. doi: 10.1186/s12876-023-02657-y (PMC9867544; doi:10.1186/s12876-023-02657-y)

	pvalue	Hazard ratio
PRR22	0.041	1.604(1.019−2.525)
STX1A	0.016	1.456(1.072−1.977)
CSAD	0.037	1.622(1.029−2.556)
RGS11	0.036	1.830(1.041−3.217)
GIGYF1	0.010	1.787(1.147−2.783)
DNASE1L2	0.026	1.678(1.064−2.648)
SHC2	0.030	1.344(1.029−1.756)
PNPLA7	0.013	1.928(1.151−3.227)
TUBGCP6	0.040	1.646(1.022−2.649)
GABBR1	0.007	1.547(1.128−2.122)
LINC00174	0.002	2.482(1.397−4.411)
TAS2R5	0.042	1.892(1.024−3.496)
HSF4	<0.001	1.775(1.276−2.470)
LTB4R	0.003	1.679(1.190−2.367)
PLXNA3	0.013	1.698(1.117−2.580)
EXOC3L4	0.003	1.882(1.241−2.856)
NLRP1	0.048	1.539(1.003−2.362)
RPL32P3	0.005	1.888(1.216−2.930)
MAN2C1	0.002	2.153(1.314−3.528)
BTBD19	0.028	1.684(1.057−2.683)
YJEFN3	0.004	1.819(1.212−2.732)
ZNF692	0.010	1.660(1.130−2.439)
LENG8	0.031	1.337(1.027−1.742)
UPK3B	<0.001	1.973(1.468−2.651)
DNAH1	0.009	2.109(1.205−3.694)
ZNF767P	0.001	1.846(1.272−2.679)
MTMR9LP	0.009	2.055(1.199−3.521)
AGAP9	<0.001	2.433(1.468−4.032)
C1RL−AS1	0.029	1.909(1.068−3.412)
TKTL1	0.012	1.280(1.056−1.552)
L3HYPDH	0.005	1.945(1.218−3.104)
ADAMTS13	0.002	2.253(1.350−3.760)
GBAP1	0.047	1.615(1.006−2.594)
CRYBB3	0.015	2.049(1.150−3.652)
GABPB1−AS1	0.036	1.418(1.023−1.965)
BRICD5	0.018	1.838(1.110−3.044)
FAM229A	0.044	1.948(1.018−3.726)
AGER	0.041	1.686(1.021−2.784)

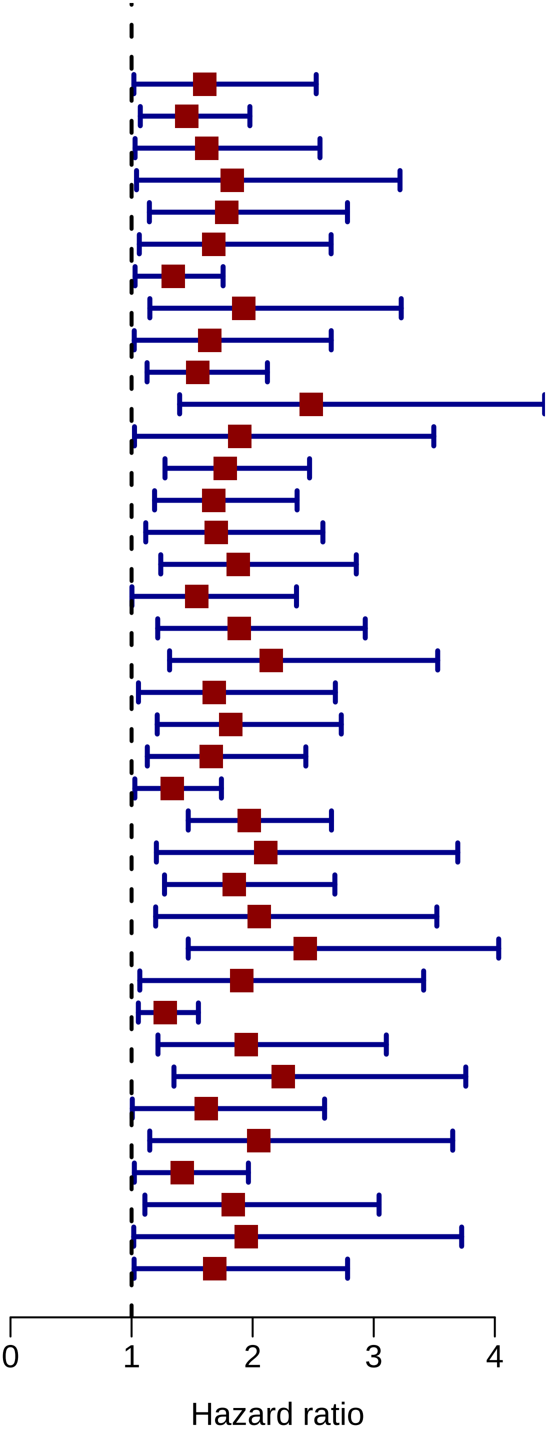

Supplement: Supplementary file 1 — Additional file 1. The original code and visual data. [file 12876_2023_2657_MOESM1_ESM.zip › 12876_2023_2657_MOESM1_ESM/14. Prognosis-related genes.zip!/14. Prognosis-related genes/20_forest.pdf]
