# Supplementary material for: Expression patterns and prognostic value of key regulators associated with m7G RNA modification based on all gene expression in colon adenocarcinoma
Source: BMC Gastroenterol. 2023 Jan 21;23:22. doi: 10.1186/s12876-023-02657-y (PMC9867544; doi:10.1186/s12876-023-02657-y)
Supplement: Supplementary file 1 — Additional file 1. The original code and visual data. [file 12876_2023_2657_MOESM1_ESM.zip › 12876_2023_2657_MOESM1_ESM/15. Prognostic model construction.zip!/15. Prognostic model construction/21_lasso.lambda.pdf]

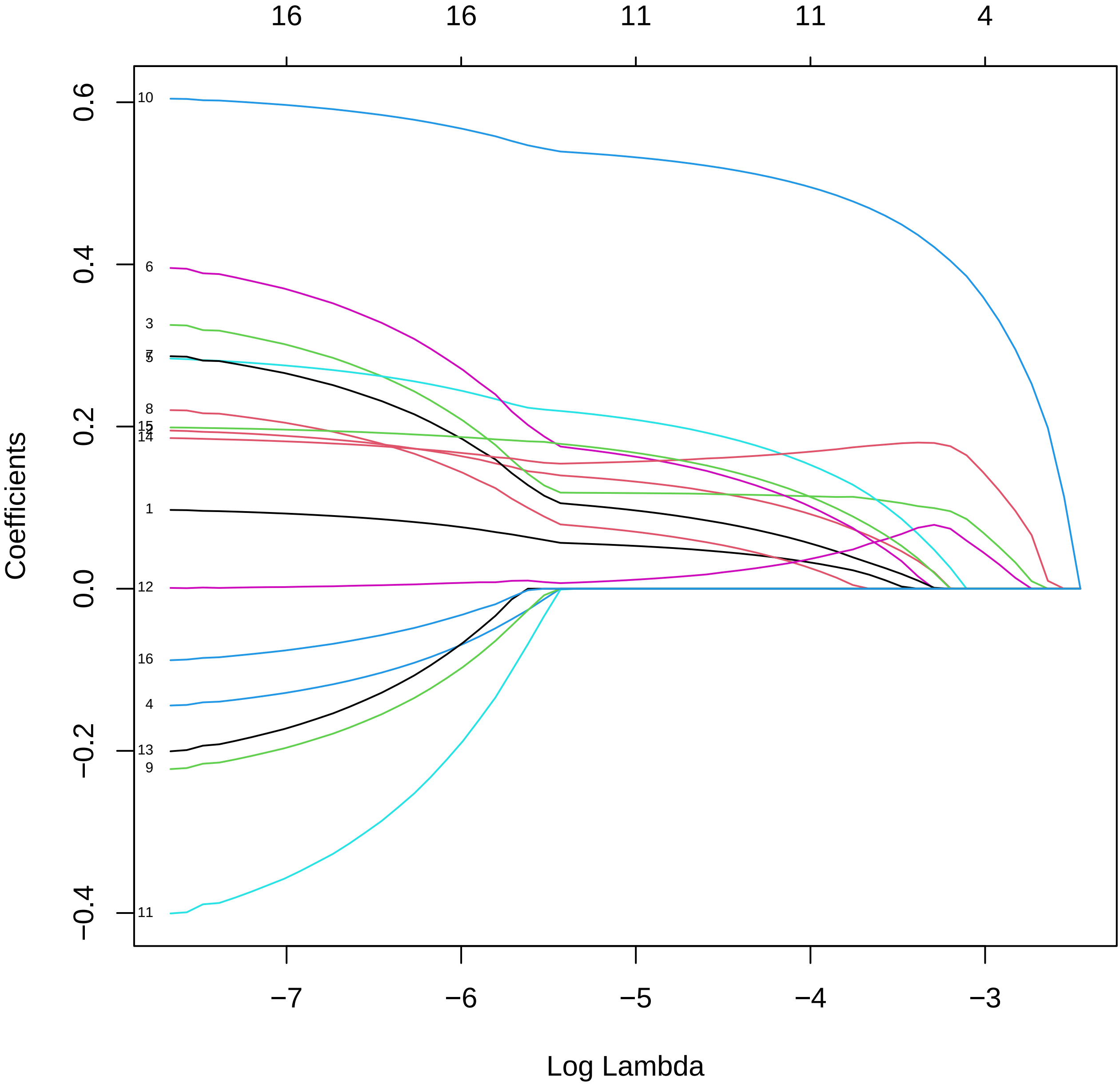

16
16
11
11
4
10
6
3
7
5
8
15
2
14
1
12
16
4
13
9
11
0.6
0.4
0.2
0.0
−0.2
−0.4
Coefficients
−7
−6
−5
−4
−3
Log Lambda
