# Supplementary material for: Expression patterns and prognostic value of key regulators associated with m7G RNA modification based on all gene expression in colon adenocarcinoma
Source: BMC Gastroenterol. 2023 Jan 21;23:22. doi: 10.1186/s12876-023-02657-y (PMC9867544; doi:10.1186/s12876-023-02657-y)
Supplement: Supplementary file 1 — Additional file 1. The original code and visual data. [file 12876_2023_2657_MOESM1_ESM.zip › 12876_2023_2657_MOESM1_ESM/16. Survival analysis.zip!/16. Survival analysis/22_testSurv.pdf]

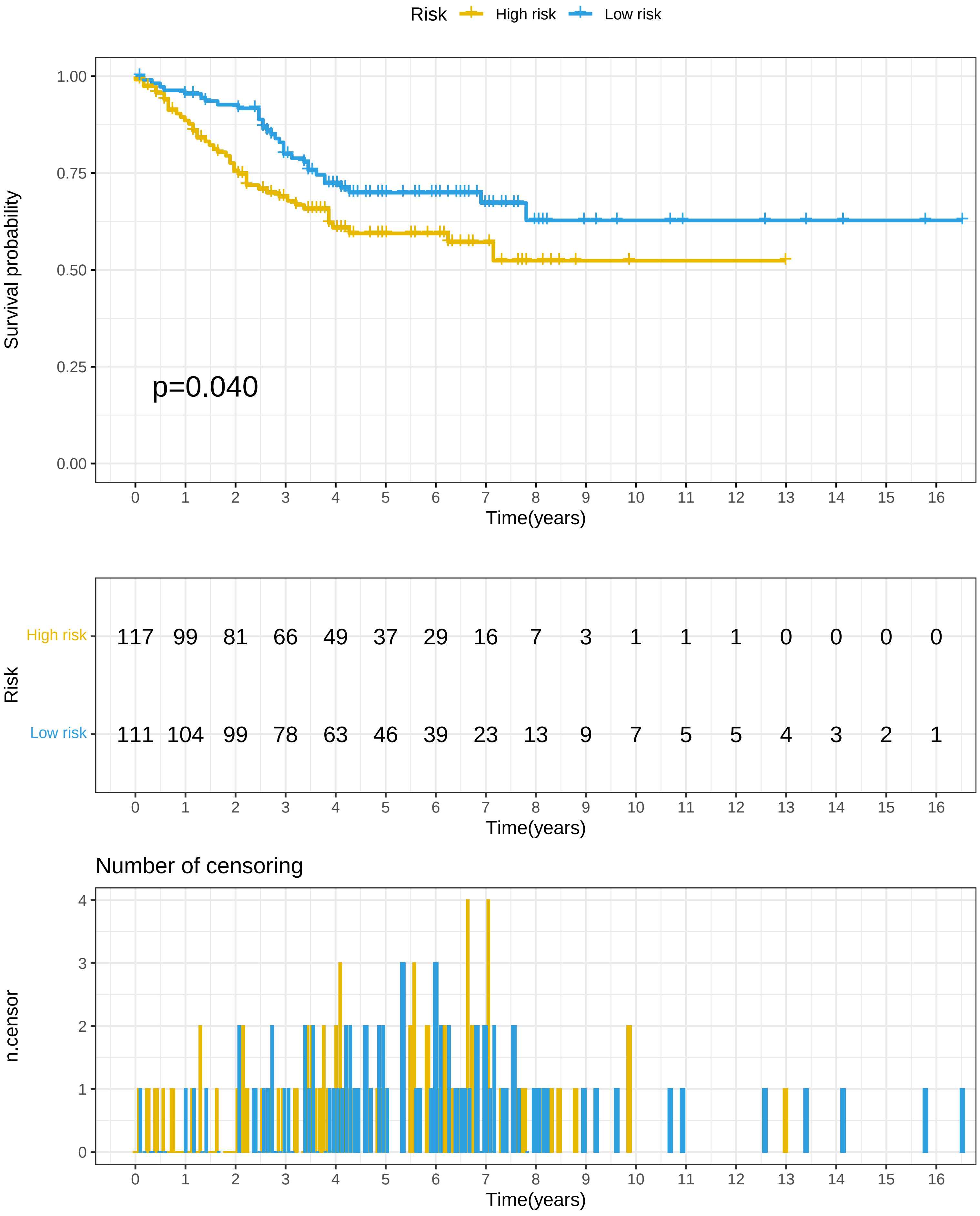
Risk
High risk
Low risk
Survival probability
1.00
0.75
0.50
0.25
0.00
p=0.040
Time(years)
0 1 2 3 4 5 6 7 8 9 10 11 12 13 14 15 16
Risk
High risk 117 99 81 66 49 37 29 16 7 3 1 1 1 0 0 0 0
Low risk 111 104 99 78 63 46 39 23 13 9 7 5 5 4 3 2 1
Time(years)
Number of censoring
n.censor
4
3
2
1
0
Time(years)
