# Supplementary material for: Expression patterns and prognostic value of key regulators associated with m7G RNA modification based on all gene expression in colon adenocarcinoma
Source: BMC Gastroenterol. 2023 Jan 21;23:22. doi: 10.1186/s12876-023-02657-y (PMC9867544; doi:10.1186/s12876-023-02657-y)
Supplement: Supplementary file 1 — Additional file 1. The original code and visual data. [file 12876_2023_2657_MOESM1_ESM.zip › 12876_2023_2657_MOESM1_ESM/16. Survival analysis.zip!/16. Survival analysis/22_trainSurv.pdf]

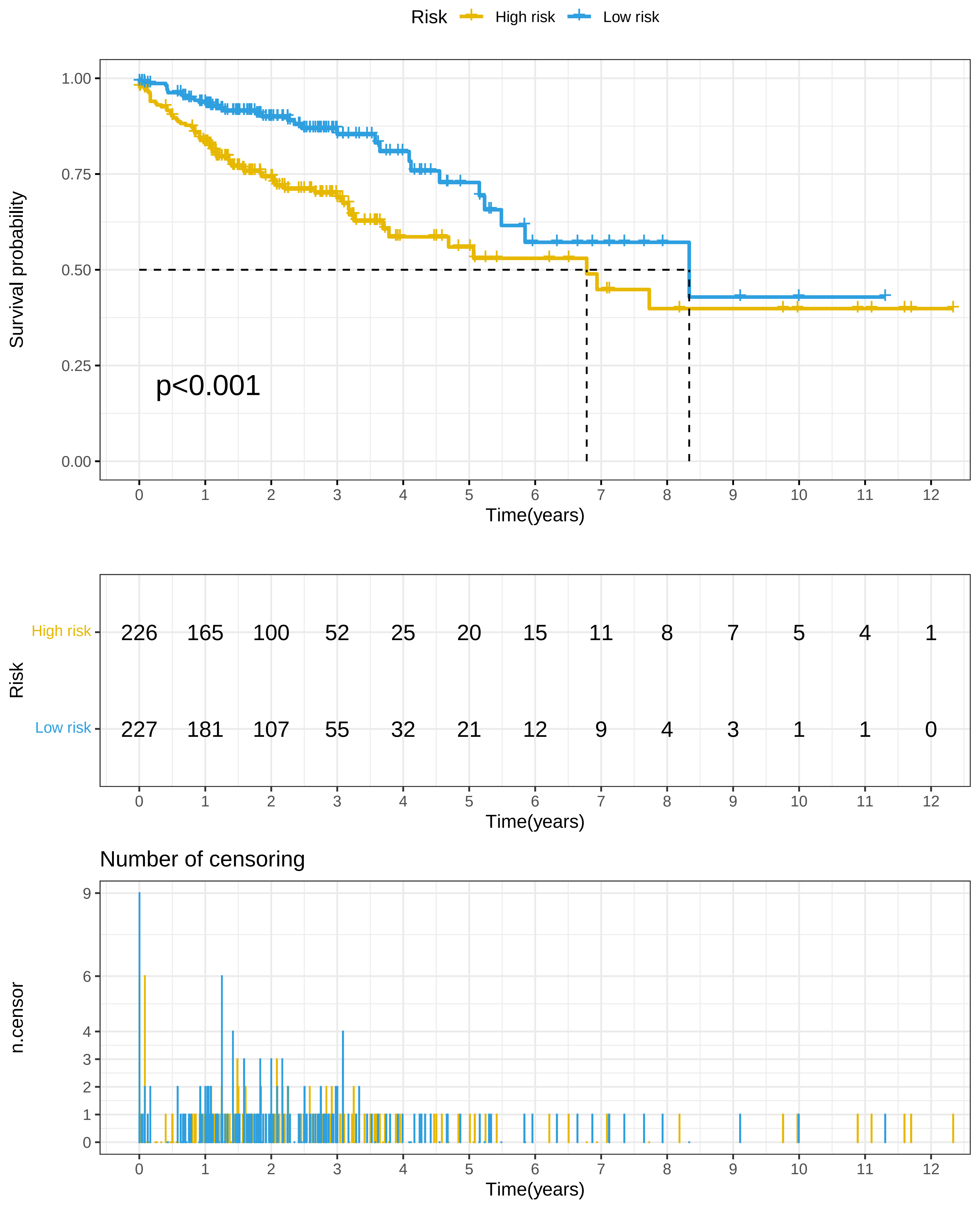
Risk
High risk
Low risk
1.00
0.75
0.50
0.25
0.00
Survival probability
p<0.001
0 1 2 3 4 5 6 7 8 9 10 11 12
Time(years)
High risk 226 165 100 52 25 20 15 11 8 7 5 4 1
Low risk 227 181 107 55 32 21 12 9 4 3 1 1 0
Risk
0 1 2 3 4 5 6 7 8 9 10 11 12
Time(years)
Number of censoring
9
6
4
3
2
1
0
n.censor
0 1 2 3 4 5 6 7 8 9 10 11 12
Time(years)
