# Supplementary material for: Expression patterns and prognostic value of key regulators associated with m7G RNA modification based on all gene expression in colon adenocarcinoma
Source: BMC Gastroenterol. 2023 Jan 21;23:22. doi: 10.1186/s12876-023-02657-y (PMC9867544; doi:10.1186/s12876-023-02657-y)
Supplement: Supplementary file 1 — Additional file 1. The original code and visual data. [file 12876_2023_2657_MOESM1_ESM.zip › 12876_2023_2657_MOESM1_ESM/17.ROC curve.zip!/17.ROC curve/23_test.ROC.pdf]

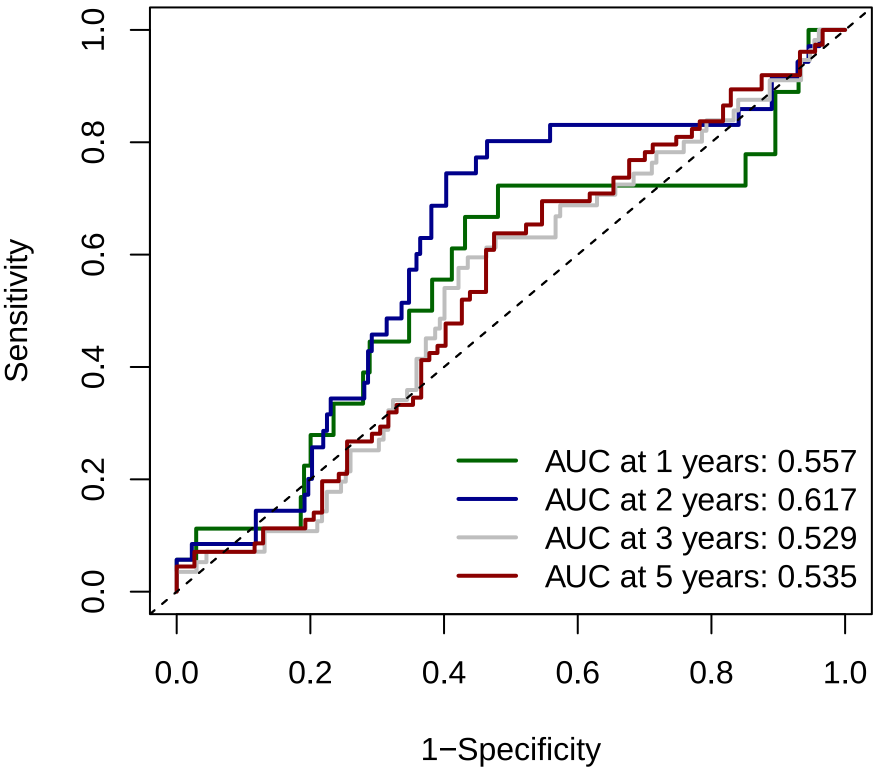

Sensitivity
1−Specificity
0.0
0.2
0.4
0.6
0.8
1.0
AUC at 1 years: 0.557
AUC at 2 years: 0.617
AUC at 3 years: 0.529
AUC at 5 years: 0.535
