# Supplementary material for: Expression patterns and prognostic value of key regulators associated with m7G RNA modification based on all gene expression in colon adenocarcinoma
Source: BMC Gastroenterol. 2023 Jan 21;23:22. doi: 10.1186/s12876-023-02657-y (PMC9867544; doi:10.1186/s12876-023-02657-y)
Supplement: Supplementary file 1 — Additional file 1. The original code and visual data. [file 12876_2023_2657_MOESM1_ESM.zip › 12876_2023_2657_MOESM1_ESM/18. Risk Curve.zip!/18. Risk Curve/24_test.riskScore.pdf]

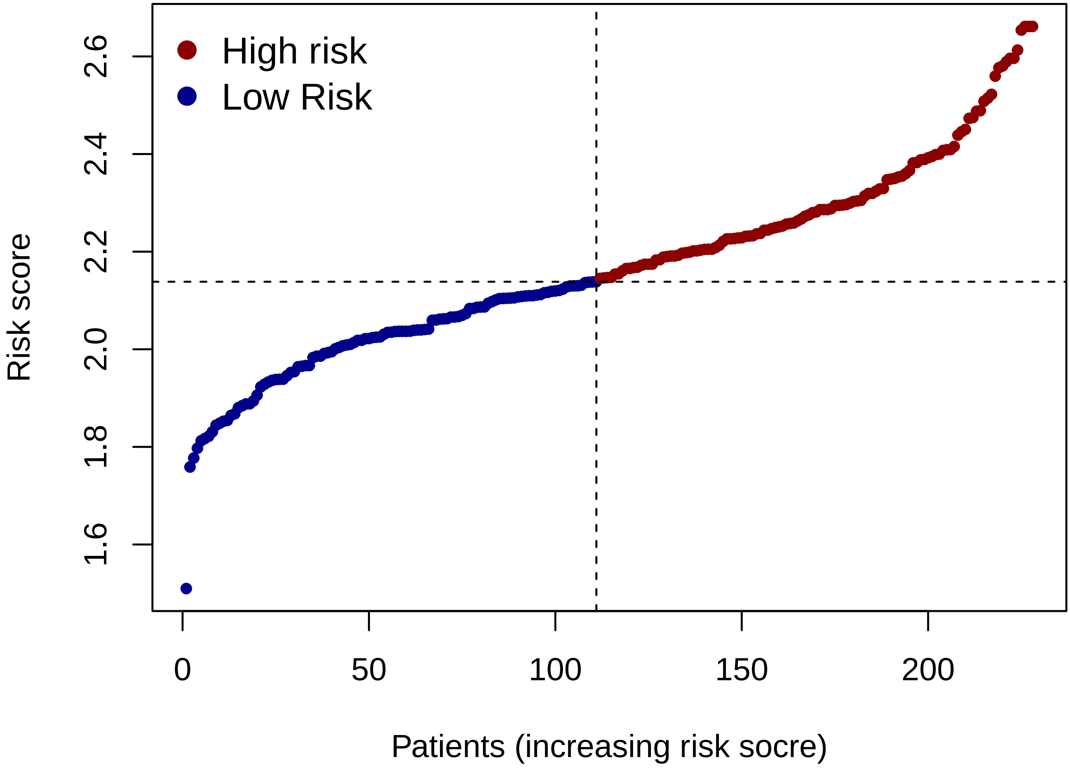
High risk
Low Risk
Risk score
2.6
2.4
2.2
2.0
1.8
1.6
0
50
100
150
200
Patients (increasing risk socre)
