# Supplementary material for: Expression patterns and prognostic value of key regulators associated with m7G RNA modification based on all gene expression in colon adenocarcinoma
Source: BMC Gastroenterol. 2023 Jan 21;23:22. doi: 10.1186/s12876-023-02657-y (PMC9867544; doi:10.1186/s12876-023-02657-y)
Supplement: Supplementary file 1 — Additional file 1. The original code and visual data. [file 12876_2023_2657_MOESM1_ESM.zip › 12876_2023_2657_MOESM1_ESM/18. Risk Curve.zip!/18. Risk Curve/24_train.survStat.pdf]

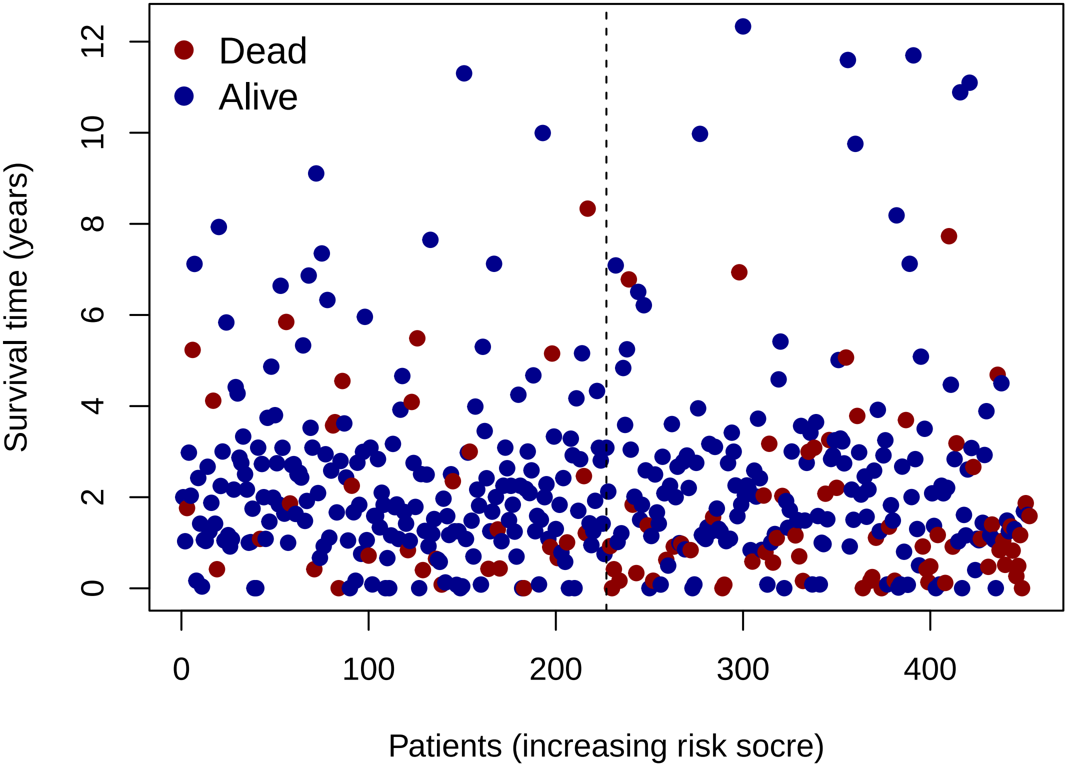

Dead
Alive
Survival time (years)
0
2
4
6
8
10
12
0
100
200
300
400
Patients (increasing risk socre)
