# Supplementary material for: Expression patterns and prognostic value of key regulators associated with m7G RNA modification based on all gene expression in colon adenocarcinoma
Source: BMC Gastroenterol. 2023 Jan 21;23:22. doi: 10.1186/s12876-023-02657-y (PMC9867544; doi:10.1186/s12876-023-02657-y)
Supplement: Supplementary file 1 — Additional file 1. The original code and visual data. [file 12876_2023_2657_MOESM1_ESM.zip › 12876_2023_2657_MOESM1_ESM/19. PCA and t-SNE analysis.zip!/19. PCA and t-SNE analysis/25_test.PCA.pdf]

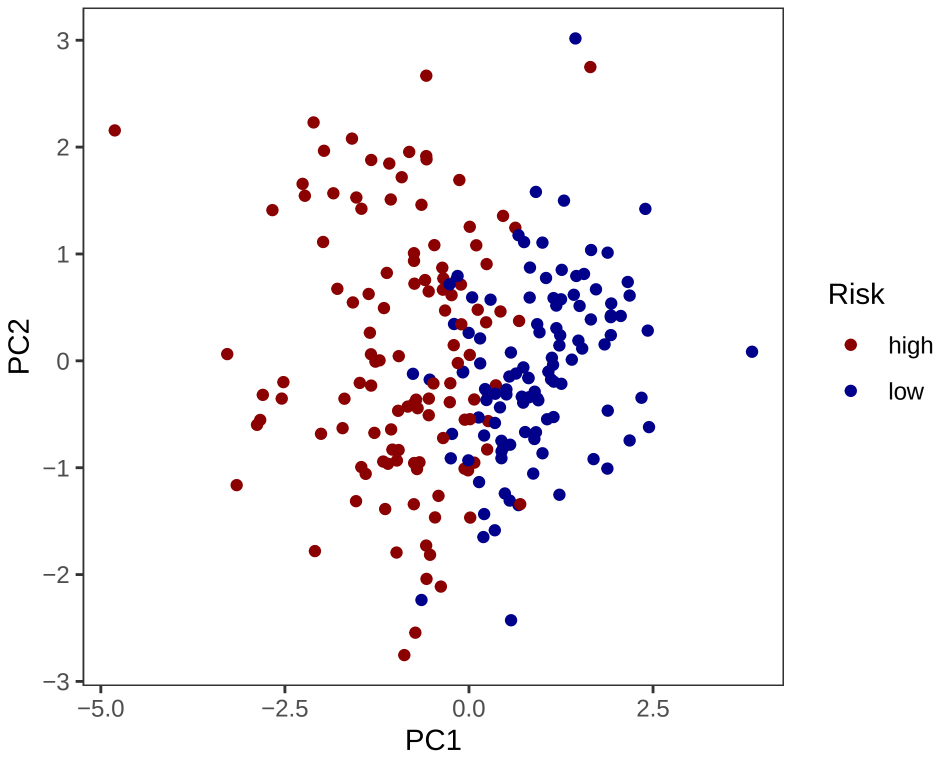

PC2
PC1
3
2
1
0
−1
−2
−3
−5.0
−2.5
0.0
2.5
Risk
high
low
