# Supplementary material for: Expression patterns and prognostic value of key regulators associated with m7G RNA modification based on all gene expression in colon adenocarcinoma
Source: BMC Gastroenterol. 2023 Jan 21;23:22. doi: 10.1186/s12876-023-02657-y (PMC9867544; doi:10.1186/s12876-023-02657-y)
Supplement: Supplementary file 1 — Additional file 1. The original code and visual data. [file 12876_2023_2657_MOESM1_ESM.zip › 12876_2023_2657_MOESM1_ESM/19. PCA and t-SNE analysis.zip!/19. PCA and t-SNE analysis/25_test.t-SNE.pdf]

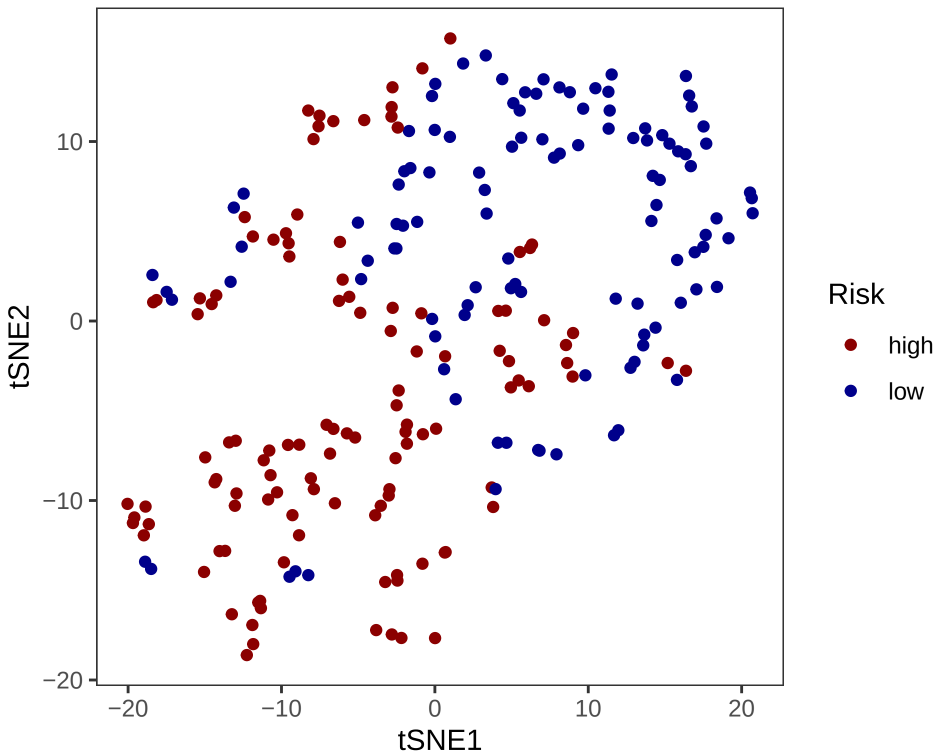

10
0
−10
−20
tSNE2
−20
−10
0
10
20
tSNE1
Risk
high
low
