# Supplementary material for: Expression patterns and prognostic value of key regulators associated with m7G RNA modification based on all gene expression in colon adenocarcinoma
Source: BMC Gastroenterol. 2023 Jan 21;23:22. doi: 10.1186/s12876-023-02657-y (PMC9867544; doi:10.1186/s12876-023-02657-y)

	pvalue	Hazard ratio
Age	<0.001	1.042(1.021−1.062)
Gender	0.424	0.837(0.541−1.295)
Stage	<0.001	2.206(1.477−3.295)
T	0.075	1.620(0.952−2.757)
N	0.885	1.029(0.703−1.504)
riskScore	<0.001	5.476(2.467−12.153)

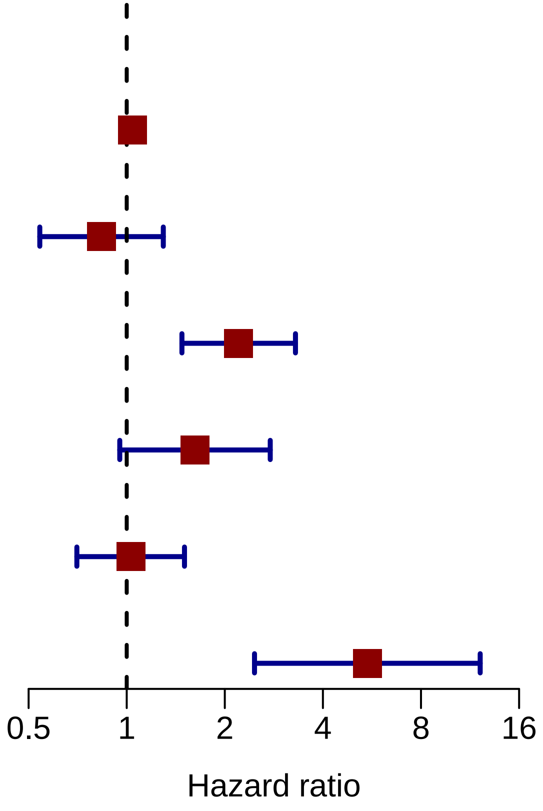

Supplement: Supplementary file 1 — Additional file 1. The original code and visual data. [file 12876_2023_2657_MOESM1_ESM.zip › 12876_2023_2657_MOESM1_ESM/20. TCGA independent prognostic analysis.zip!/20. TCGA independent prognostic analysis/26_multiForest.pdf]
