# Supplementary material for: Expression patterns and prognostic value of key regulators associated with m7G RNA modification based on all gene expression in colon adenocarcinoma
Source: BMC Gastroenterol. 2023 Jan 21;23:22. doi: 10.1186/s12876-023-02657-y (PMC9867544; doi:10.1186/s12876-023-02657-y)
Supplement: Supplementary file 1 — Additional file 1. The original code and visual data. [file 12876_2023_2657_MOESM1_ESM.zip › 12876_2023_2657_MOESM1_ESM/20. TCGA independent prognostic analysis.zip!/20. TCGA independent prognostic analysis/26_uniForest.pdf]

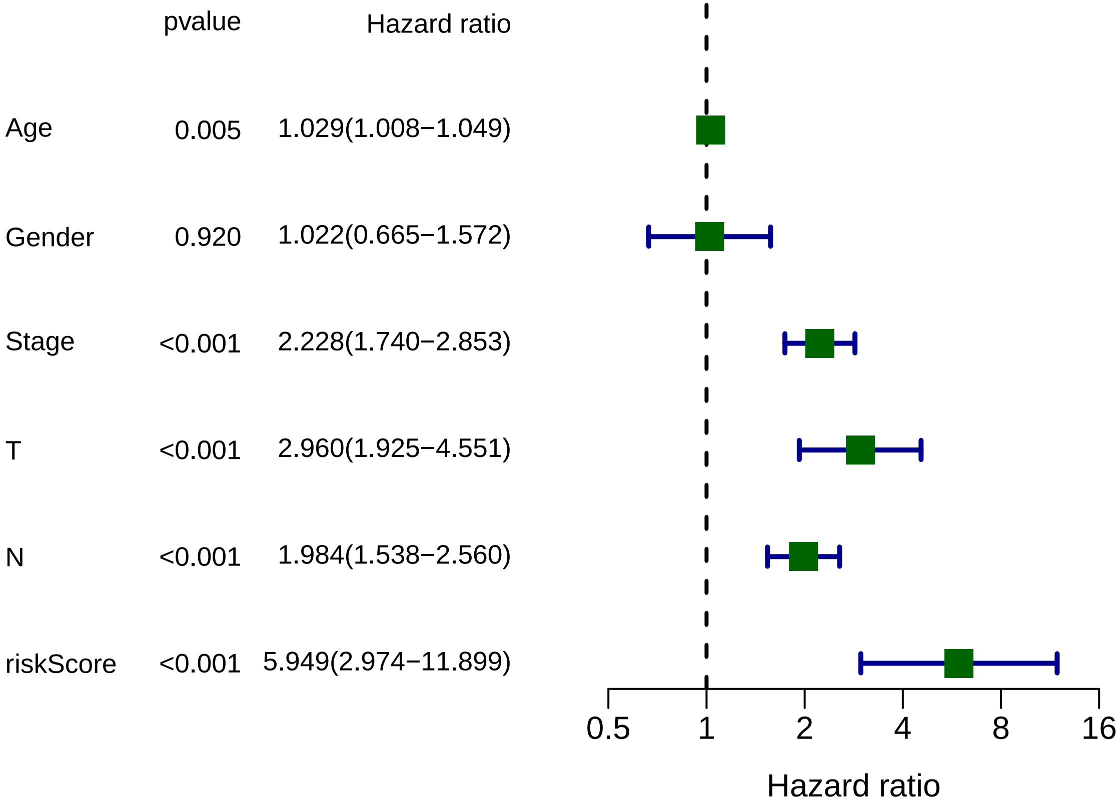

pvalue
Hazard ratio
Age
0.005
1.029(1.008−1.049)
Gender
0.920
1.022(0.665−1.572)
Stage
<0.001
2.228(1.740−2.853)
T
<0.001
2.960(1.925−4.551)
N
<0.001
1.984(1.538−2.560)
riskScore
<0.001
5.949(2.974−11.899)
0.5
1
2
4
8
16
Hazard ratio
