# Supplementary material for: Expression patterns and prognostic value of key regulators associated with m7G RNA modification based on all gene expression in colon adenocarcinoma
Source: BMC Gastroenterol. 2023 Jan 21;23:22. doi: 10.1186/s12876-023-02657-y (PMC9867544; doi:10.1186/s12876-023-02657-y)
Supplement: Supplementary file 1 — Additional file 1. The original code and visual data. [file 12876_2023_2657_MOESM1_ESM.zip › 12876_2023_2657_MOESM1_ESM/21. Risk Heat Map.zip!/21. Risk Heat Map/27_heatmap.pdf]

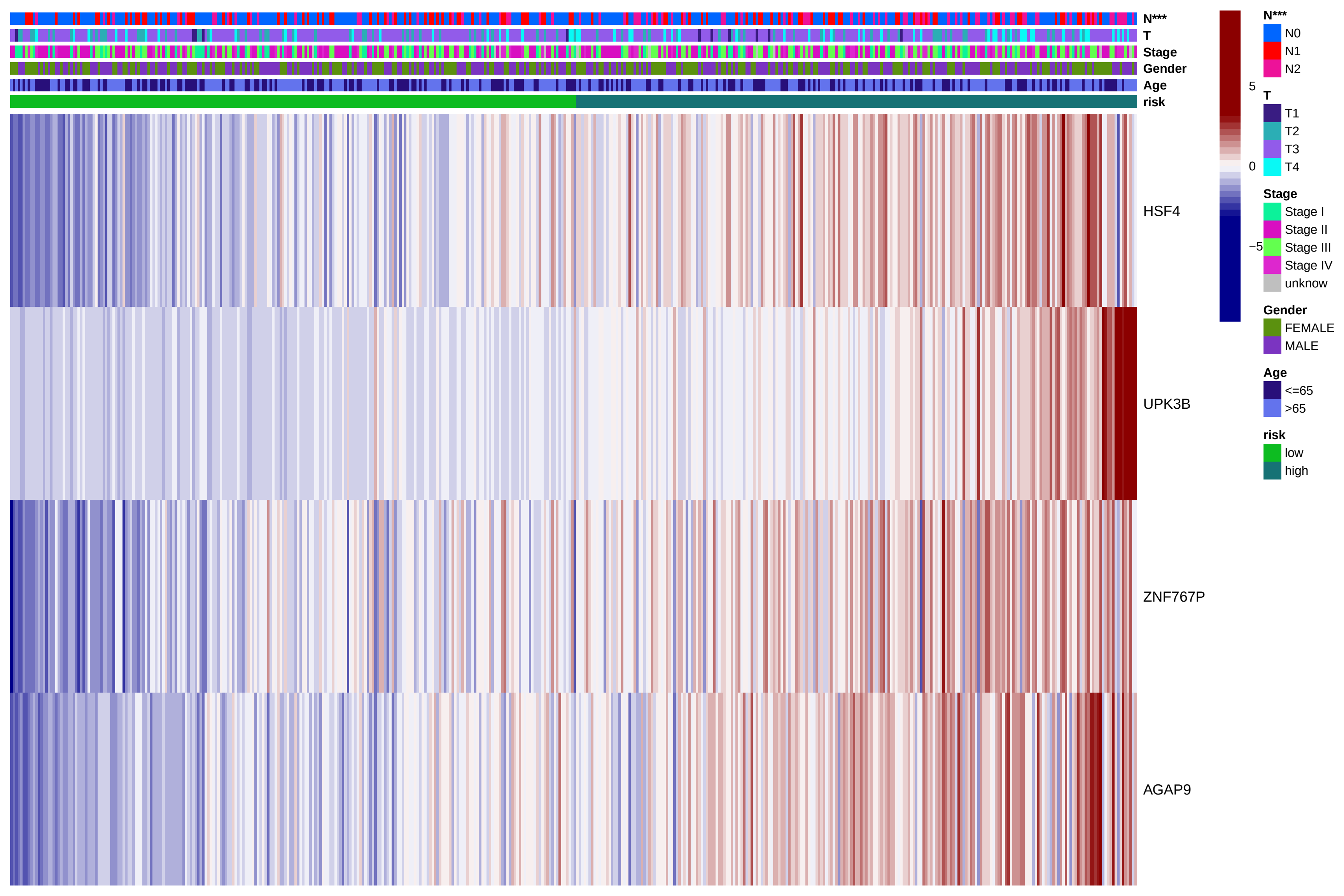
N***
T
Stage
Gender
Age
risk
HSF4
UPK3B
ZNF767P
AGAP9
5
0
−5
N***
N0
N1
N2
T
T1
T2
T3
T4
Stage
Stage I
Stage II
Stage III
Stage IV
unknow
Gender
FEMALE
MALE
Age
<=65
>65
risk
low
high
