# Supplementary material for: Expression patterns and prognostic value of key regulators associated with m7G RNA modification based on all gene expression in colon adenocarcinoma
Source: BMC Gastroenterol. 2023 Jan 21;23:22. doi: 10.1186/s12876-023-02657-y (PMC9867544; doi:10.1186/s12876-023-02657-y)
Supplement: Supplementary file 1 — Additional file 1. The original code and visual data. [file 12876_2023_2657_MOESM1_ESM.zip › 12876_2023_2657_MOESM1_ESM/23.GO enrichment analysis.zip!/23.GO enrichment analysis/29_GOHistogram.pdf]

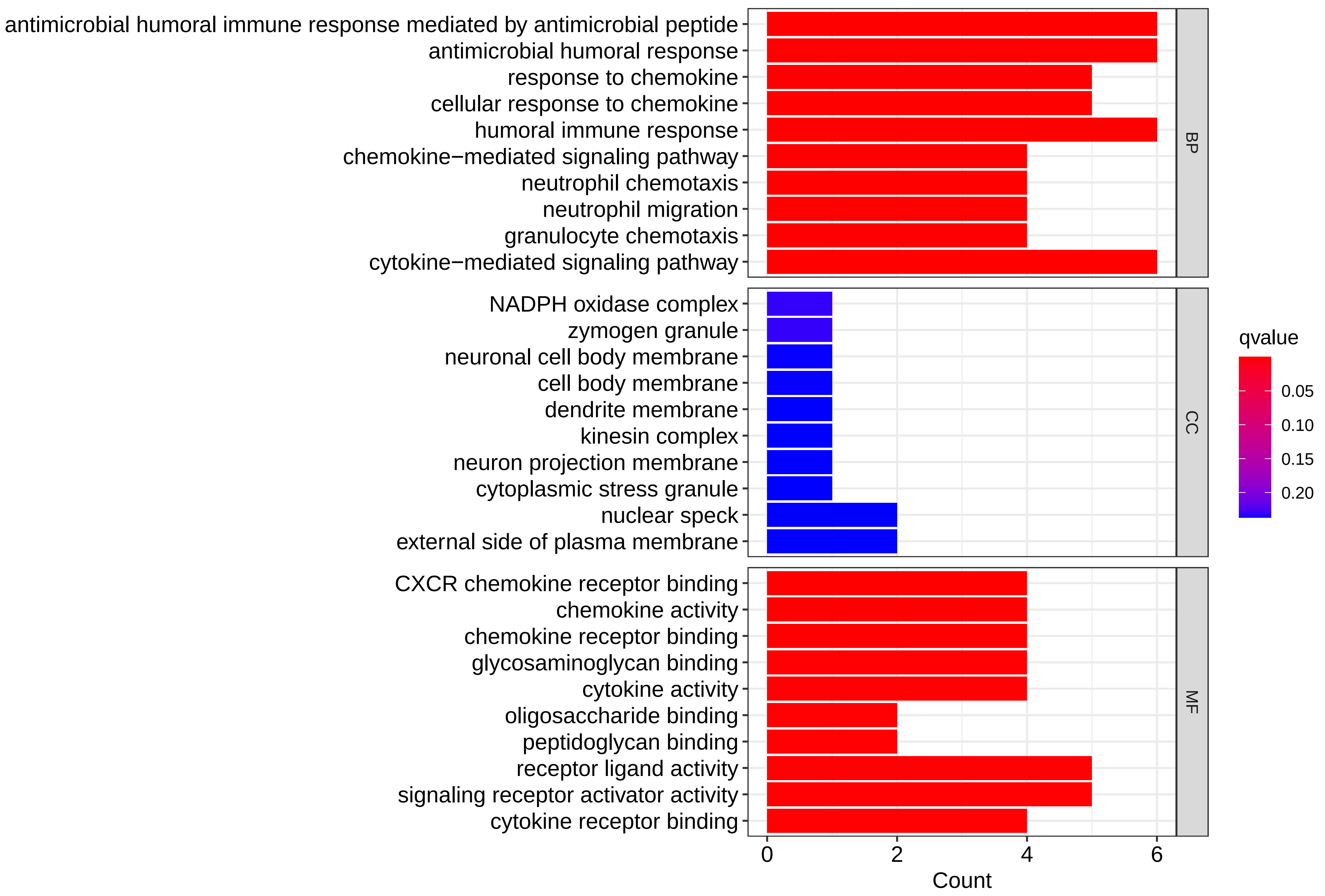
antimicrobial humoral immune response mediated by antimicrobial peptide
antimicrobial humoral response
response to chemokine
cellular response to chemokine
humoral immune response
chemokine−mediated signaling pathway
neutrophil chemotaxis
neutrophil migration
granulocyte chemotaxis
cytokine−mediated signaling pathway
BP
NADPH oxidase complex
zymogen granule
neuronal cell body membrane
cell body membrane
dendrite membrane
kinesin complex
neuron projection membrane
cytoplasmic stress granule
nuclear speck
external side of plasma membrane
CC
CXCR chemokine receptor binding
chemokine activity
chemokine receptor binding
glycosaminoglycan binding
cytokine activity
oligosaccharide binding
peptidoglycan binding
receptor ligand activity
signaling receptor activator activity
cytokine receptor binding
MF
0
2
4
6
Count
qvalue
0.05
0.10
0.15
0.20
