# Supplementary material for: Expression patterns and prognostic value of key regulators associated with m7G RNA modification based on all gene expression in colon adenocarcinoma
Source: BMC Gastroenterol. 2023 Jan 21;23:22. doi: 10.1186/s12876-023-02657-y (PMC9867544; doi:10.1186/s12876-023-02657-y)
Supplement: Supplementary file 1 — Additional file 1. The original code and visual data. [file 12876_2023_2657_MOESM1_ESM.zip › 12876_2023_2657_MOESM1_ESM/23.GO enrichment analysis.zip!/23.GO enrichment analysis/29_GOcircle diagram.pdf]

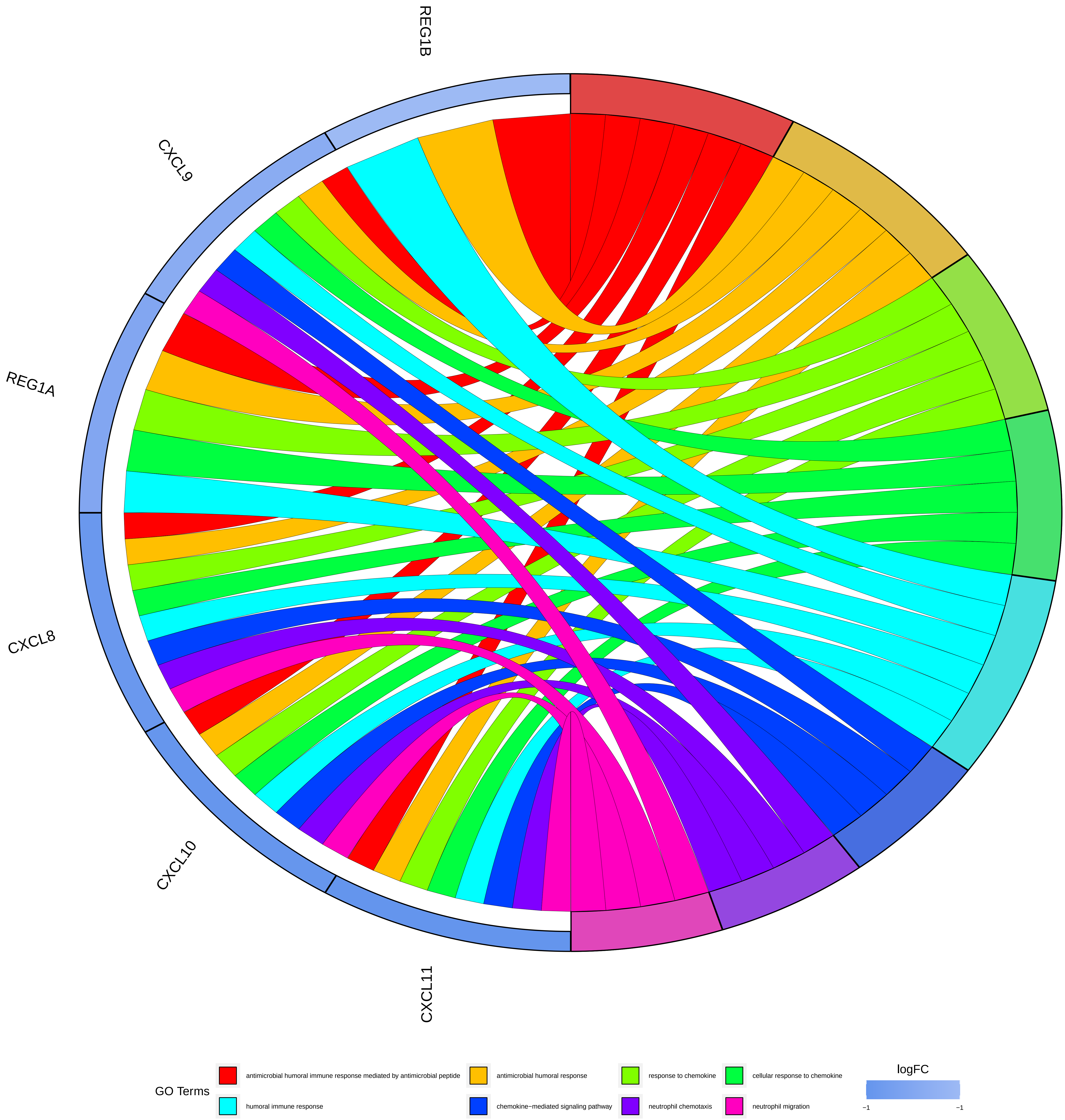
REG1B
CXCL9
REG1A
CXCL8
CXCL10
CXCL11
GO Terms
antimicrobial humoral immune response mediated by antimicrobial peptide
antimicrobial humoral response
response to chemokine
cellular response to chemokine
humoral immune response
chemokine−mediated signaling pathway
neutrophil chemotaxis
neutrophil migration
logFC
−1
−1
