# Supplementary material for: Expression patterns and prognostic value of key regulators associated with m7G RNA modification based on all gene expression in colon adenocarcinoma
Source: BMC Gastroenterol. 2023 Jan 21;23:22. doi: 10.1186/s12876-023-02657-y (PMC9867544; doi:10.1186/s12876-023-02657-y)
Supplement: Supplementary file 1 — Additional file 1. The original code and visual data. [file 12876_2023_2657_MOESM1_ESM.zip › 12876_2023_2657_MOESM1_ESM/24.kegg enrichment analysis.R.zip!/24.kegg enrichment analysis.R/30_KEGGcircle diagram.pdf]

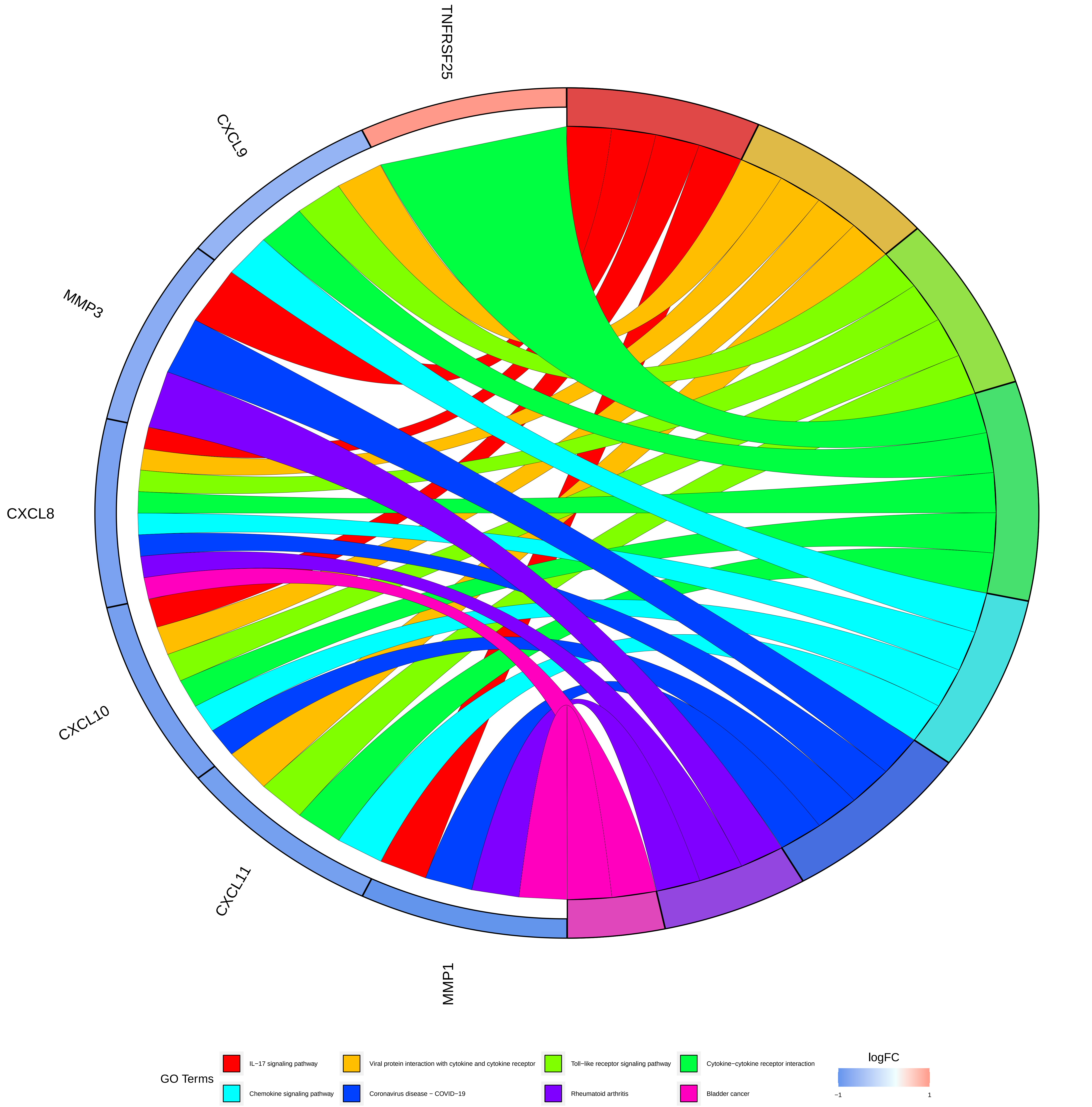

TNFRSF25
CXCL9
MMP3
CXCL8
CXCL10
CXCL11
MMP1
GO Terms
IL−17 signaling pathway
Viral protein interaction with cytokine and cytokine receptor
Toll−like receptor signaling pathway
Cytokine−cytokine receptor interaction
Chemokine signaling pathway
Coronavirus disease − COVID−19
Rheumatoid arthritis
Bladder cancer
logFC
−1
1
