# Supplementary material for: Expression patterns and prognostic value of key regulators associated with m7G RNA modification based on all gene expression in colon adenocarcinoma
Source: BMC Gastroenterol. 2023 Jan 21;23:22. doi: 10.1186/s12876-023-02657-y (PMC9867544; doi:10.1186/s12876-023-02657-y)
Supplement: Supplementary file 1 — Additional file 1. The original code and visual data. [file 12876_2023_2657_MOESM1_ESM.zip › 12876_2023_2657_MOESM1_ESM/24.kegg enrichment analysis.R.zip!/24.kegg enrichment analysis.R/30_keggHistogram.pdf]

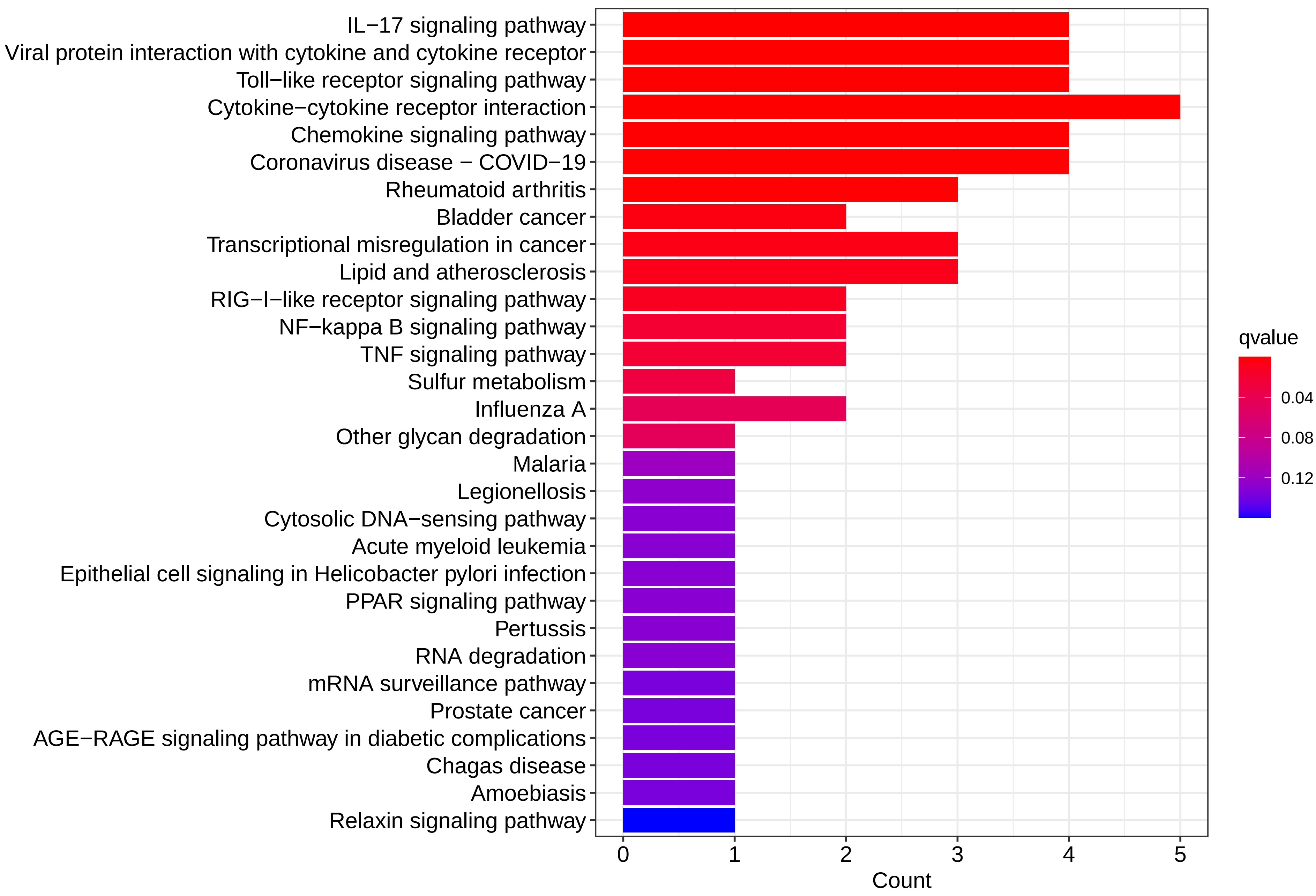
IL−17 signaling pathway
Viral protein interaction with cytokine and cytokine receptor
Toll−like receptor signaling pathway
Cytokine−cytokine receptor interaction
Chemokine signaling pathway
Coronavirus disease − COVID−19
Rheumatoid arthritis
Bladder cancer
Transcriptional misregulation in cancer
Lipid and atherosclerosis
RIG−I−like receptor signaling pathway
NF−kappa B signaling pathway
TNF signaling pathway
Sulfur metabolism
Influenza A
Other glycan degradation
Malaria
Legionellosis
Cytosolic DNA−sensing pathway
Acute myeloid leukemia
Epithelial cell signaling in Helicobacter pylori infection
PPAR signaling pathway
Pertussis
RNA degradation
mRNA surveillance pathway
Prostate cancer
AGE−RAGE signaling pathway in diabetic complications
Chagas disease
Amoebiasis
Relaxin signaling pathway
0
1
2
3
4
5
Count
qvalue
0.04
0.08
0.12
