# Supplementary material for: Expression patterns and prognostic value of key regulators associated with m7G RNA modification based on all gene expression in colon adenocarcinoma
Source: BMC Gastroenterol. 2023 Jan 21;23:22. doi: 10.1186/s12876-023-02657-y (PMC9867544; doi:10.1186/s12876-023-02657-y)
Supplement: Supplementary file 1 — Additional file 1. The original code and visual data. [file 12876_2023_2657_MOESM1_ESM.zip › 12876_2023_2657_MOESM1_ESM/26. Immunodifferential analysis.zip!/26. Immunodifferential analysis/GEO.immFunction.pdf]

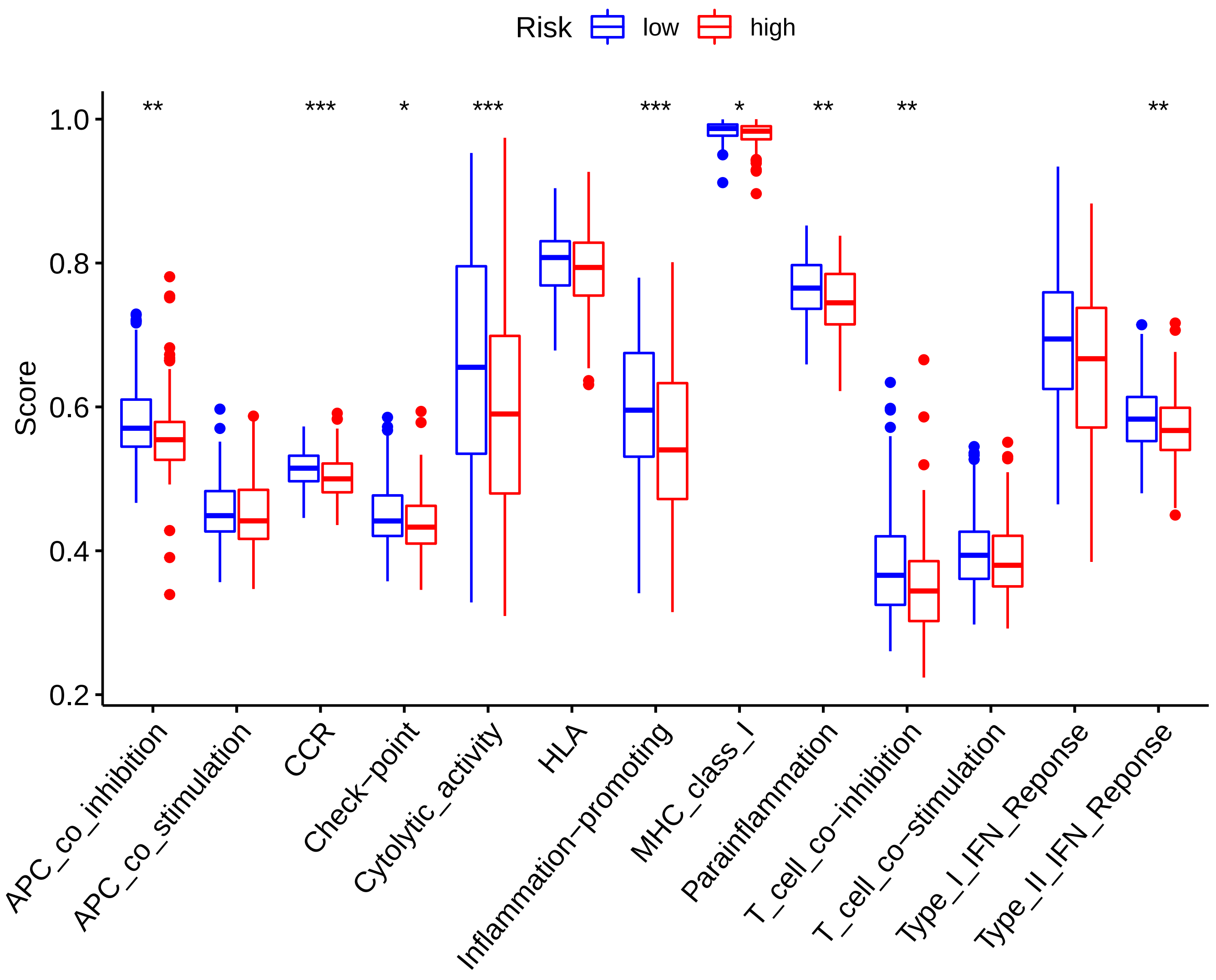
Risk
low
high
Score
1.0
0.8
0.6
0.4
0.2
**
***
*
***
***
*
**
**
**
APC_co_inhibition
APC_co_stimulation
CCR
Check−point
Cytolytic_activity
HLA
Inflammation−promoting
MHC_class_I
Parainflammation
T_cell_co−inhibition
T_cell_co−stimulation
Type_I_IFN_Reponse
Type_II_IFN_Reponse
