# Supplementary material for: Expression patterns and prognostic value of key regulators associated with m7G RNA modification based on all gene expression in colon adenocarcinoma
Source: BMC Gastroenterol. 2023 Jan 21;23:22. doi: 10.1186/s12876-023-02657-y (PMC9867544; doi:10.1186/s12876-023-02657-y)
Supplement: Supplementary file 1 — Additional file 1. The original code and visual data. [file 12876_2023_2657_MOESM1_ESM.zip › 12876_2023_2657_MOESM1_ESM/27. Nomotsu.zip!/27. Nomotsu/Rplot.pdf]

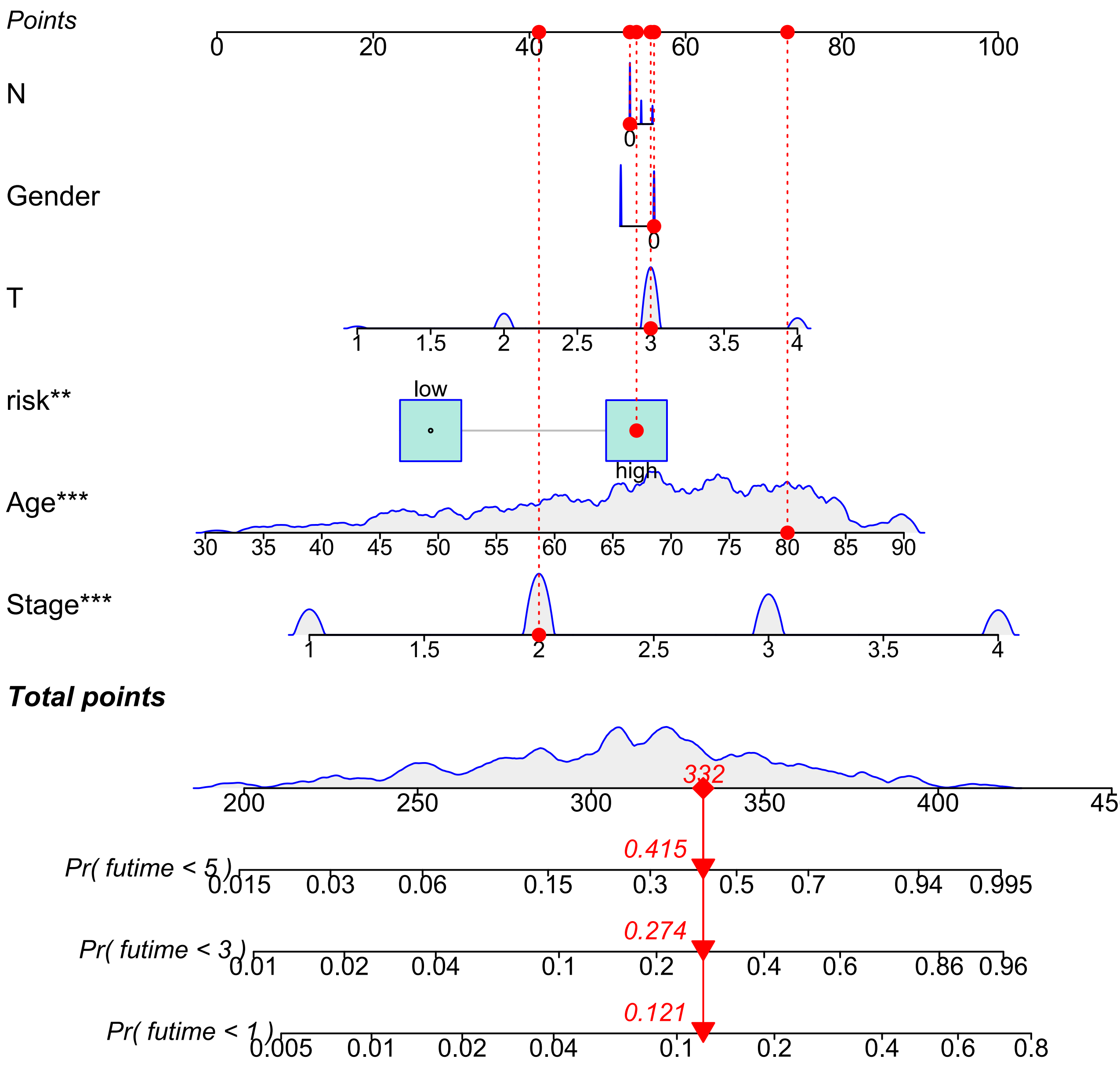

Points
0 20 40 60 80 100
N
0
Gender
0
T
1 1.5 2 2.5 3 3.5 4
risk**
low
high
Age***
30 35 40 45 50 55 60 65 70 75 80 85 90
Stage***
1 1.5 2 2.5 3 3.5 4
Total points
332
200 250 300 350 400 45
Pr(futime < 5)
0.415
0.015 0.03 0.06 0.15 0.3 0.5 0.7 0.94 0.995
Pr(futime < 3)
0.274
0.01 0.02 0.04 0.1 0.2 0.4 0.6 0.86 0.96
Pr(futime < 1)
0.121
0.005 0.01 0.02 0.04 0.1 0.2 0.4 0.6 0.8
