# Supplementary material for: Expression patterns and prognostic value of key regulators associated with m7G RNA modification based on all gene expression in colon adenocarcinoma
Source: BMC Gastroenterol. 2023 Jan 21;23:22. doi: 10.1186/s12876-023-02657-y (PMC9867544; doi:10.1186/s12876-023-02657-y)
Supplement: Supplementary file 1 — Additional file 1. The original code and visual data. [file 12876_2023_2657_MOESM1_ESM.zip › 12876_2023_2657_MOESM1_ESM/5. m7G gene box line diagram.zip!/5. m7G gene box line diagram/11_M7G.boxplot.pdf]

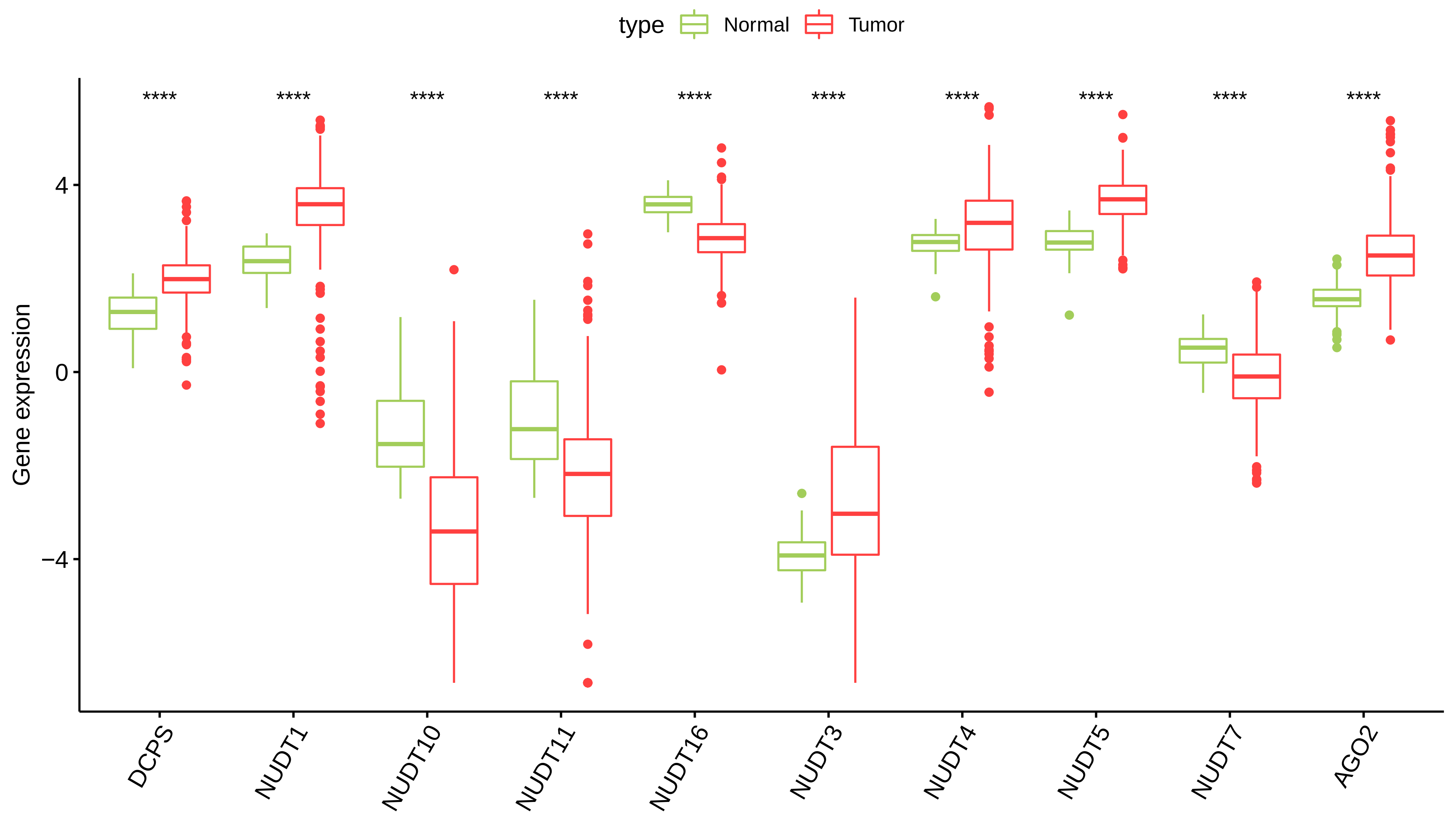
type
Normal
Tumor
Gene expression
4
0
−4
****
DCPS
NUDT1
NUDT10
NUDT11
NUDT16
NUDT3
NUDT4
NUDT5
NUDT7
AGO2
