# Supplementary material for: Expression patterns and prognostic value of key regulators associated with m7G RNA modification based on all gene expression in colon adenocarcinoma
Source: BMC Gastroenterol. 2023 Jan 21;23:22. doi: 10.1186/s12876-023-02657-y (PMC9867544; doi:10.1186/s12876-023-02657-y)
Supplement: Supplementary file 1 — Additional file 1. The original code and visual data. [file 12876_2023_2657_MOESM1_ESM.zip › 12876_2023_2657_MOESM1_ESM/6. m7G gene correlation heat map.zip!/6. m7G gene correlation heat map/12_m7G gene correlation heat map.pdf]

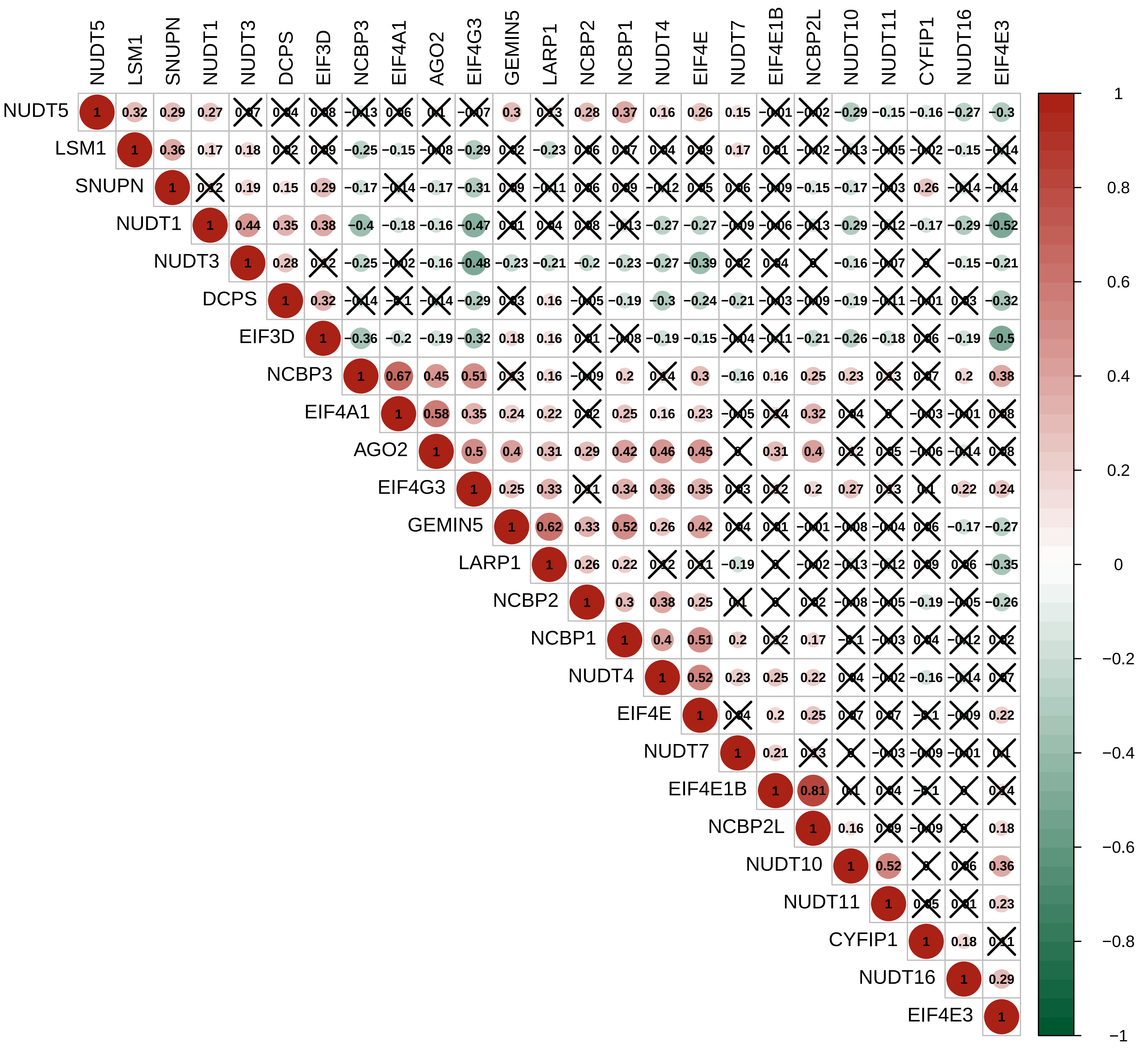
NUDT5
LSM1
SNUPN
NUDT1
NUDT3
DCPS
EIF3D
NCBP3
EIF4A1
AGO2
EIF4G3
GEMIN5
LARP1
NCBP2
NCBP1
NUDT4
EIF4E
NUDT7
EIF4E1B
NCBP2L
NUDT10
NUDT11
CYFIP1
NUDT16
EIF4E3
1
0.8
0.6
0.4
0.2
0
−0.2
−0.4
−0.6
−0.8
−1
