# Supplementary material for: Expression patterns and prognostic value of key regulators associated with m7G RNA modification based on all gene expression in colon adenocarcinoma
Source: BMC Gastroenterol. 2023 Jan 21;23:22. doi: 10.1186/s12876-023-02657-y (PMC9867544; doi:10.1186/s12876-023-02657-y)
Supplement: Supplementary file 1 — Additional file 1. The original code and visual data. [file 12876_2023_2657_MOESM1_ESM.zip › 12876_2023_2657_MOESM1_ESM/8. survival typing analysis.zip!/8. survival typing analysis/14_survival.pdf]

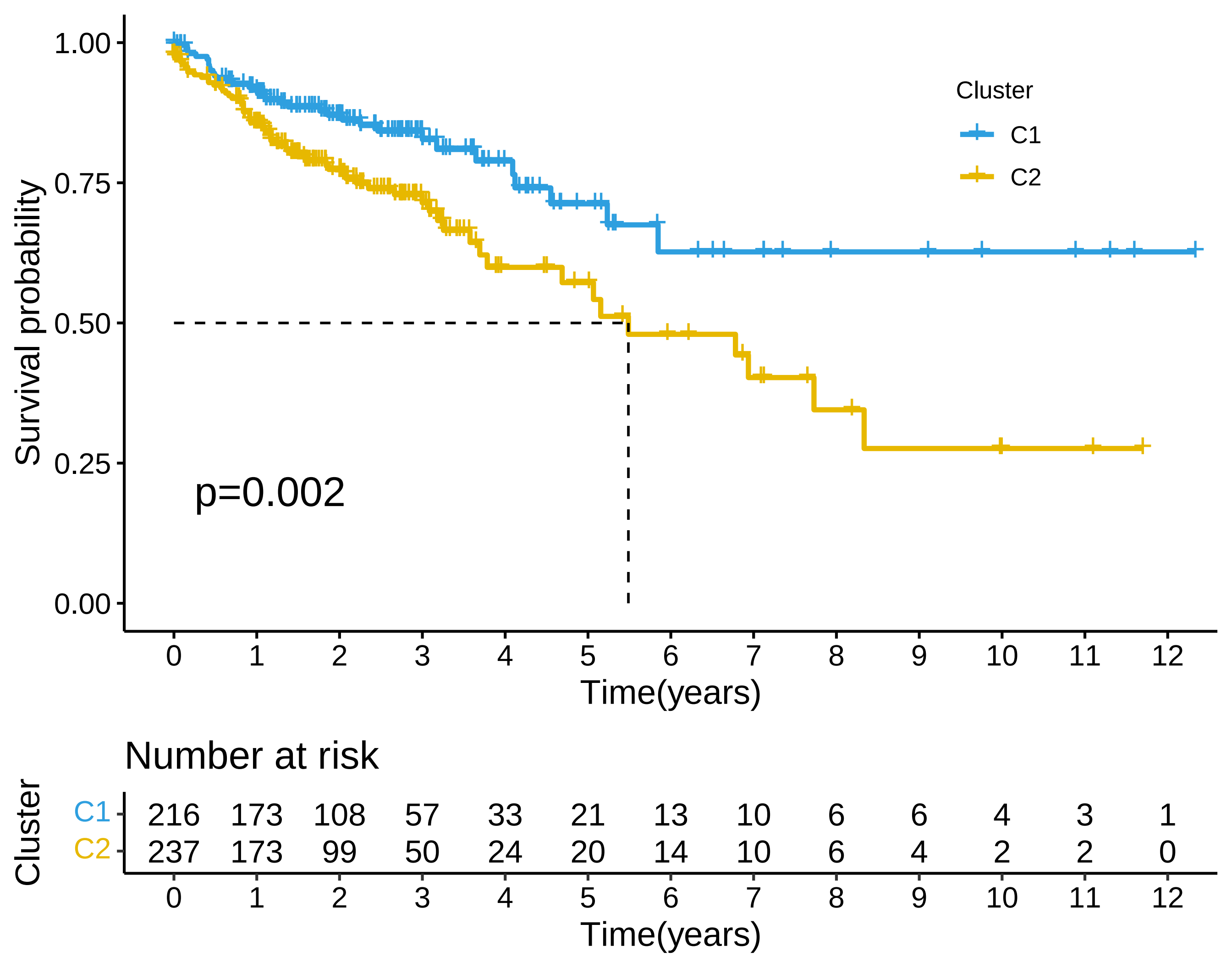
Survival probability
1.00
0.75
0.50
0.25
0.00
p=0.002
Cluster
C1
C2
0 1 2 3 4 5 6 7 8 9 10 11 12
Time(years)
Number at risk
Cluster
C1 216 173 108 57 33 21 13 10 6 6 4 3 1
C2 237 173 99 50 24 20 14 10 6 4 2 2 0
0 1 2 3 4 5 6 7 8 9 10 11 12
Time(years)
