# Supplementary material for: Expression patterns and prognostic value of key regulators associated with m7G RNA modification based on all gene expression in colon adenocarcinoma
Source: BMC Gastroenterol. 2023 Jan 21;23:22. doi: 10.1186/s12876-023-02657-y (PMC9867544; doi:10.1186/s12876-023-02657-y)
Supplement: Supplementary file 1 — Additional file 1. The original code and visual data. [file 12876_2023_2657_MOESM1_ESM.zip › 12876_2023_2657_MOESM1_ESM/R package reference address.docx]

**BiocManager**: Morgan M (2022). _BiocManager: Access the Bioconductor Project Package Repository. R package version 1.30.19,<https://CRAN.R-project.org/package=BiocManager>.

**limma:** Ritchie, M.E., Phipson, B., Wu, D., Hu, Y., Law, C.W., Shi, W., and Smyth, G.K. (2015). limma powers differential expression analyses for RNA-sequencing and microarray studies. Nucleic Acids Research 43(7), e47.

**pheatmap:** Kolde R (2019). pheatmap: Pretty Heatmaps. R package version 1.0.12, <https://CRAN.R-project.org/package=pheatmap>.

**ggpubr:** Kassambara A (2020). _ggpubr: 'ggplot2' Based Publication Ready Plots_. R package version 0.4.0, <https://CRAN.R-project.org/package=ggpubr>.

**corrplot:** Taiyun Wei and Viliam Simko (2021). R package 'corrplot': Visualization of Correlation Matrix (Version 0.92). Available from <https://github.com/taiyun/corrplot>

**survival:** Therneau T (2022). A Package for Survival Analysis in R. R package version 3.4-0,<https://CRAN.R-project.org/package=survival>.

**survminer:** Kassambara A, Kosinski M, Biecek P (2021). survminer:Drawing Survival Curves using 'ggplot2'. R package version 0.4.9, <https://CRAN.R-project.org/package=survminer>.

**ggplot2:**H. Wickham. ggplot2: Elegant Graphics for Data Analysis. Springer-Verlag New York, 2016.

**pheatmap**: Kolde R (2019). _pheatmap: Pretty Heatmaps_. R package version 1.0.12, <https://CRAN.R-project.org/package=pheatmap>.

**colorspace:** Stauffer R, Mayr GJ, Dabernig M, Zeileis A (2009).“Somewhere over the Rainbow: How to Make Effective Use of Colors in Meteorological Visualizations.”Bulletin of the American Meteorological Society_, *96*(2), 203-216. doi:10.1175/BAMS-D-13-00155.1

<https://doi.org/10.1175/BAMS-D-13-00155.1>.

**stringi :**Gagolewski M (2022). “stringi: Fast and portable character string processing in R.” Journal of Statistical Software_, *103*(2), 1-59. doi:10.18637/jss.v103.i02 <https://doi.org/10.18637/jss.v103.i02>.

**digest:** Lucas DEwcbA, Tuszynski J, Bengtsson H, Urbanek S, Frasca M, Lewis B, Stokely M, Muehleisen H, Murdoch D, Hester J, Wu W, Kou Q, Onkelinx T, Lang M, Simko V, Hornik K, Neal

R, Bell K, de Queljoe M, Suruceanu I, Denney B, Schumacher D, Chang. aW (2021). _digest: Create Compact Hash Digests of R Objects_. R package version 0.6.29, <https://CRAN.R-project.org/package=digest>.

**ggpubr:** Kassambara A (2020). _ggpubr: 'ggplot2' Based Publication Ready Plots_. R package version 0.4.0, <https://CRAN.R-project.org/package=ggpubr>.

**regplot:** Marshall R (2020). _regplot: Enhanced Regression Nomogram Plot_. R package version 1.1, <https://CRAN.R-project.org/package=regplot>.
