# Supplementary material for: Expression patterns and prognostic value of key regulators associated with m7G RNA modification based on all gene expression in colon adenocarcinoma
Source: BMC Gastroenterol. 2023 Jan 21;23:22. doi: 10.1186/s12876-023-02657-y (PMC9867544; doi:10.1186/s12876-023-02657-y)
Supplement: Supplementary file 1 — Additional file 1. The original code and visual data. [file 12876_2023_2657_MOESM1_ESM.zip › 12876_2023_2657_MOESM1_ESM/Table S1.docx]

| Name of gene | Primer sequences（5'to3') |
| --- | --- |
| HSF4-F(homo) | GGCAAGCTGATCCAGTGTCT |
| HSF4-R(homo) | GCAGGTGTTGAACTTGGCAG |
| UPK3B-F(homo) | TCGTCATTACCTCCATCCTCTC |
| UPK3B-R(homo) | GATGTGGTGGGTCATGTAGC |
| ZNF767P-F(homo) | AACCTCAACCTCCTCCTACC |
| ZNF767P-R(homo) | CTTGTCCTGCTGGGTCATTT |
| AGAP9-F(homo) | AGGAGGTGAACGAGACCTGT |
| AGAP9-R(homo) | ATGACGTCCACCCCGTCAG |
| GAPDH-F(homo） | GGTGTGAACCATGAGAAGTATGA |
| GAPDH-R(homo） | GAGTCCTTCCACGATACCAAAG |
